# Systematic Review and Meta-Analysis of the Impact of Bariatric Surgery on Future Cancer Risk

**DOI:** 10.3390/ijms24076192

**Published:** 2023-03-24

**Authors:** Robert B. Wilson, Dhruvi Lathigara, Devesh Kaushal

**Affiliations:** 1 Department of Upper Gastrointestinal Surgery, University of New South Wales, Liverpool Hospital, Liverpool, NSW 2170, Australia; 2 Department of General Surgery, University of Western Sydney, Campbelltown Hospital, Campbelltown, NSW 2560, Australia

**Keywords:** bariatric surgery, cancer, obesity, metabolic surgery, exosome, cytokine, oestrogen, diabetes, adipokine, leptin, GLP-1, semaglutide, metabolic syndrome, weight loss, carcinogen, sleeve gastrectomy, gastric bypass surgery, NASH, meta-analysis

## Abstract

The study aimed to perform a systematic review and meta-analysis of the evidence for the prevention of future cancers following bariatric surgery. A systematic literature search of the Cochrane Library, Embase, Scopus, Web of Science and PubMed databases (2007–2023), Google Scholar and grey literature was conducted. A meta-analysis was performed using the inverse variance method and random effects model. Thirty-two studies involving patients with obesity who received bariatric surgery and control patients who were managed with conventional treatment were included. The meta-analysis suggested bariatric surgery was associated with a reduced overall incidence of cancer (RR 0.62, 95% CI 0.46–0.84, *p* < 0.002), obesity-related cancer (RR 0.59, 95% CI 0.39–0.90, *p* = 0.01) and cancer-associated mortality (RR 0.51, 95% CI 0.42–0.62, *p* < 0.00001). In specific cancers, bariatric surgery was associated with reduction in the future incidence of hepatocellular carcinoma (RR 0.35, 95% CI 0.22–0.55, *p* < 0.00001), colorectal cancer (RR 0.63, CI 0.50–0.81, *p* = 0.0002), pancreatic cancer (RR 0.52, 95% CI 0.29–0.93, *p* = 0.03) and gallbladder cancer (RR 0.41, 95% CI 0.18–0.96, *p* = 0.04), as well as female specific cancers, including breast cancer (RR 0.56, 95% CI 0.44–0.71, *p* < 0.00001), endometrial cancer (RR 0.38, 95% CI 0.26–0.55, *p* < 0.00001) and ovarian cancer (RR 0.45, 95% CI 0.31–0.64, *p* < 0.0001). There was no significant reduction in the incidence of oesophageal, gastric, thyroid, kidney, prostate cancer or multiple myeloma after bariatric surgery as compared to patients with morbid obesity who did not have bariatric surgery. Obesity-associated carcinogenesis is closely related to metabolic syndrome; visceral adipose dysfunction; aromatase activity and detrimental cytokine, adipokine and exosomal miRNA release. Bariatric surgery results in long-term weight loss in morbidly obese patients and improves metabolic syndrome. Bariatric surgery may decrease future overall cancer incidence and mortality, including the incidence of seven obesity-related cancers.

## 1. Introduction

Obesity is emerging as a global health problem [1,2,3,4,5,6,7,8,9,10]. It was once thought to be more prevalent in affluent Western nations but is now rapidly increasing in North African, Middle Eastern, Central and South American, South and South-East Asian and Oceanic countries [2,3]. Obesity is closely associated with the genesis and potentiation of cancer. In 2013, 4.3% of all cancers in Australia were attributable to obesity, with global estimates ranging from 4.5% in the UK to 20% in the United States [3,4]. In Australia in 2021, cancer accounted for nearly one-fifth (18%) of the total disease burden, with an estimated 162,000 new cancer cases diagnosed in 2022 [1].

Obesity is the predominant contributor to Australia’s non-fatal burden of disease. In 2017–2018, nearly two-thirds of the Australian adult population and one-quarter of children and adolescents were classified as either obese (body mass index (BMI) ≥ 30 kg/m^2^) or overweight (BMI = 25.0 to 29.9 kg/m^2^). There were proportionately more adult males than females who were overweight (42.0% vs. 29.6%) or with Class I obesity (BMI = 30–34.9 kg/m^2^) (21.7% vs. 17.7%). There were less adult males than females with Class II obesity (BMI = 35–39.9 kg/m^2^) (7.6% vs. 7.8%) or Class III obesity (BMI ≥ 40 kg/m^2^) (3.3% vs. 4.7%) [5].

Obesity and the associated metabolic syndrome are key risk factors for multiple chronic conditions such as hyperlipidaemia, diabetes, cardiovascular disease, hypertension, obstructive sleep apnoea (OSA), non-alcoholic steatohepatitis (NASH) and some cancers. A 2014 UK population-based study and a 2011 review demonstrated a linear association between each 5 kg/m^2^ increase in BMI and an increased risk of uterine, gallbladder, kidney, cervical, thyroid cancers and leukaemia [6,7]. In 2016, the International Agency for Research on Cancer (IARC) identified thirteen different types of cancer as overweight or obesity-associated cancers (OACs): postmenopausal breast cancer, endometrial cancer, ovarian cancer, hepatocellular cancer, colorectal cancer, pancreatic cancer, adenocarcinoma of the oesophagus, gastric cardia cancer, gallbladder cancer, kidney (renal cell) cancer, thyroid cancer, multiple myeloma and meningioma. There were eight other cancers for which the evidence for an association was thought to be inadequate due to limited data or inconsistent results: cancers of the lung, oesophagus (squamous cell carcinoma), gastric non-cardia, extrahepatic biliary tract, skin (cutaneous melanoma), testis, urinary bladder and brain or spinal cord (glioma) [8].

Of the 10 most common cancers in Australia in 2022, breast cancer (2nd), colorectal (4th), kidney (7th), pancreatic (8th) and uterine cancer (10th) were the OACs with the highest estimated incidence of diagnosis [1]. When mortality was examined based on tumour histology, the OACs associated with the highest estimated number of deaths in 2022 were ranked as colorectal (2nd), pancreatic (3rd), breast (5th), liver (7th) and oesophageal adenocarcinoma (10th) [1]. There has also been a pronounced rise in Australia in age-specific cancer rates across all adult age groups below 50 years for both breast and colorectal cancers [1]. For instance, the colorectal cancer incidence rate in people aged 20–29 years increased from 4.4 in 2001 to an estimated 10.3 cases per 100,000 people in 2021, which may be attributable to the rising prevalence of obesity [1]. A recent ecological study reported an increase in the incidence of OACs by birth cohort across all age groups in Australia, which has been globally mirrored [9].

This is especially evident in the United States population, where 40% of the nearly 1.6 million cancers diagnosed (55% of the 799,734 cancers among women and 24% of the 796,752 cancers among males) in 2014 were OACs. Much of this sex difference in cancer incidence was due to endometrial, ovarian and postmenopausal female breast cancers accounting for 42% (268,091) of overweight- and obesity-related cancers. However, in cancers affecting both males and females, the incidence rates in 2014 were higher among males than females for colorectal cancer (44.1 per 100,000 vs. 33.7 per 100,000), kidney cancer (20.9 vs. 10.6), pancreatic cancer (14.4 vs. 11.1), liver cancer (11.2 vs. 3.4), adenocarcinoma of the oesophagus (5.4 vs. 0.8), multiple myeloma (7.5 vs. 4.9) and gastric cardia cancer (3.6 vs. 0.8). Females had higher rates than males of thyroid cancer (21.3 vs. 7.4) and gallbladder cancer (1.4 vs. 0.8) [10].

It was estimated that the number of colorectal cancers prevented by screening programs in the USA between 2005 and 2014 (224,800) was offset by 211,800 excess cases from other overweight- and obesity-related cancers in the same time period. When colorectal cancer was excluded from overweight- and obesity-related cancers, a 7% increase in the overall incidence of OAC was observed. Adenocarcinoma of the oesophagus, gastric cardia, liver or kidneys was nearly twice as likely in overweight persons or obese persons than healthy weight persons (BMI = 18.5–24.9 kg/m^2^) and endometrial cancer two to four times more likely in overweight/obese women. Colorectal cancer was 30% less likely to develop in persons of healthy weight than unhealthy weight persons [10].

The molecular mechanisms involved in obesity and carcinogenesis are closely related [11,12,13,14,15,16,17,18,19,20,21,22]. These include hyperinsulinemia, elevated leptin, chronic inflammation, oxidative stress, activation of HIF-1α and cytokines, DNA methylation, dysfunctional visceral adipose tissue secretome, adipokine and exosome miRNA release and alterations in the sex hormone metabolism. There is a likely dietary component, including the consumption of highly processed foods, a high-fat diet and exposure to exogenous and endogenously formed carcinogens. Obesity contributes to the development of metabolic syndrome, mainly due to increased central adiposity [18]. This can be measured by the waist circumference, waist-to-hip circumference ratio or visceral fat volume. Metabolic syndrome is present if a patient has three or more of the following criteria: abdominal obesity, high blood pressure, impaired fasting glycaemic control, high plasma triglyceride levels and low plasma HDL cholesterol levels [18]. The rising global prevalence of obesity, metabolic syndrome and obesity-associated cancers emphasises the need for further investigation, screening and effective obesity interventions [18].

Conventional obesity management usually consists of dietary modification, caloric restriction, weight reduction and exercise. However, such lifestyle measures result in only modest weight loss, ranging, at most, 5–10% of an individual’s total body weight (TBW) [23,24]. Lifestyle interventions have poor sustainability due to an established hypothalamic ‘set point’ and compensatory neurohormonal responses to attempted caloric restriction during dieting and increased energy expenditure during exercise. This ultimately conserves weight or promotes weight regain in obese patients who attempt to lose weight [25,26,27,28]. Other factors limiting the efficacy of conventional treatments include persistent systemic inflammation, OSA, a dysfunctional adipocyte secretome and metabolic syndrome in patients with morbid obesity, as well as individual comorbidities such as poor mobility, osteoarthritis, depression, sarcopenia or micronutrient deficiencies [25]. 

Bariatric surgery is an effective and robust weight loss intervention indicated for:

1. patients with a BMI ≥ 40 kg/m^2^, with or without coexisting comorbidities (Class III obesity)
2. patients with a BMI = 35–39.9 kg/m^2^ (Class II obesity) and at least one severe obesity-related comorbidity.
3. patients with a BMI = 30–34.9 kg/m^2^ (Class I obesity) and type II diabetes mellitus (T2DM) with inadequate glycaemic control, despite an optimal lifestyle and medical therapy [29].

This is according to the updated 2019 clinical guidelines by the American Association of Clinical Endocrinologists and the American Society for Metabolic and Bariatric Surgery. An additional guideline recommendation included adjusting the BMI criteria for bariatric procedures for ethnicity: 18.5–22.9 kg/m^2^ defines normal range, 23–24.9 kg/m^2^ overweight and ≥25 kg/m^2^ obesity for Asian individuals [29]. Bariatric metabolic surgery (BMS), including laparoscopic Roux-en-Y gastric bypass (RYGB), sleeve gastrectomy (LSG) and duodenal switch (DS)/biliopancreatic diversion (BPD) procedures, is particularly effective in treating obese patients with metabolic syndrome [18].

Bariatric metabolic surgery alters the gastrointestinal anatomy and the gut endocrine system, resulting in rapid postoperative improvements in glucose metabolism, diabetes, leptin sensitivity and appetite that are independent from weight loss [24,30]. LSG restricts the volume of food that patients can consume, and also decreases gastric ghrelin secretion by removal of the fundus. Ghrelin is an orexigenic hormone that normally acts on the hypothalamus and hippocampus orexigenic neurons to increase appetite and food intake and decrease metabolic activity and fat catabolism in humans [31].

RYGB results in rapid emptying of the smaller gastric pouch, which accelerates the passage of nutrients. Rapid gastric emptying is also associated with decreased gastric volume and accommodation after LSG. Rapid nutrient delivery to the small intestines results in increased satiety and decreased food reward by promoting the release of intestinal hormones such as cholecystokinin (CCK), peptide YY, glucagon-like peptide-1 (GLP-1) and oxyntomodulin. The accelerated nutrient passage promotes the release of the incretins GLP-1 and glucose-dependent insulinotropic polypeptide (GIP), which increases insulin release and lowers postprandial glycaemia [30]. In addition, there are changes in the gut microbiome and bile salt physiology, which can be enhanced by high fibre, plant-based diets after BMS [24].

Bariatric metabolic surgery can result in sustained weight loss in obese patients, while improving insulin resistance, T2DM (92% remission rate), dyslipidaemia (76% remission rate), OSA (96% remission rate), inflammation, hypoxia, oxidative stress, non-alcoholic fatty liver disease (NAFLD) and cardiovascular risk factors (58% remission rate) [32]. Consequently, observational studies have suggested a reduction in cancer incidence following bariatric surgery for morbid obesity, particularly in future gynaecological malignancies (endometrial, ovarian and breast cancer) but also in some non-hormone-associated cancers [33].

The aim of this systematic review and meta-analysis was to examine the effect of bariatric surgery on the prevention of future hormone-sensitive female specific cancers but also investigate whether bariatric surgery reduced the incidence of all obesity-associated cancers, non-hormone-related cancers, overall cancers or cancer-related mortality.

## 2. Methods

The systematic review and meta-analysis were performed according to the PRISMA guidelines to evaluate the impact of bariatric surgery on future cancer risks [34,35]. Following the PRISMA recommendations, we selected a specific population (P), intervention (I), comparator (C), outcome (O) and study design (S) (PICOS) framework to define the study eligibility:

Population (P): adult individuals (>18 years old), diagnosed with morbid obesity, followed-up for at least 3 years to investigate the incidence of cancer;
Intervention (I): bariatric surgery;
Comparison (C): simple observation or any behavioural or medical treatment;
Outcomes (O): risk of developing a cancer during the follow-up period;
Study design (S): retrospective and prospective comparative studies with at least 10 patients per group [34].

### 2.1. Search Strategy

A systematic literature search of electronic databases Cochrane Library, Embase, Scopus, Web of Science and PubMed (MEDLINE), as well as Google Scholar and grey literature, was conducted by two independent reviewers. The following search terms were used to search all databases: ‘bariatric surgery’, ‘gastric bypass’, ‘roux-en-y’, ‘gastric sleeve’, ‘sleeve gastrectomy’, ‘cancer’, ‘neoplasm’, ‘neoplasms’, ‘incidence’, ‘epidemiology’ and ‘epidemiologic studies’. The search terms were used in conjunction with the medical subject headings (MeSH): ‘Bariatric surgery’, ‘Neoplasm’, ‘Incidence’, ‘Epidemiology’ and ‘Epidemiological Studies’. Searches were performed with the Boolean operators AND or OR: (“bariatric surgery” OR “gastric bypass” OR “roux-en-y” OR “gastric sleeve” OR “sleeve gastrectomy”) AND (cancer OR neoplasm OR neoplasms) AND (incidence OR epidemiology OR “epidemiologic studies” OR “cancer epidemiology” OR “cancer incidence” OR “event rate”). Additional studies were sourced from the reference lists of relevant studies. The search was limited to studies in English from 2007 to the present.

### 2.2. Study Selection

**Figure 1:**
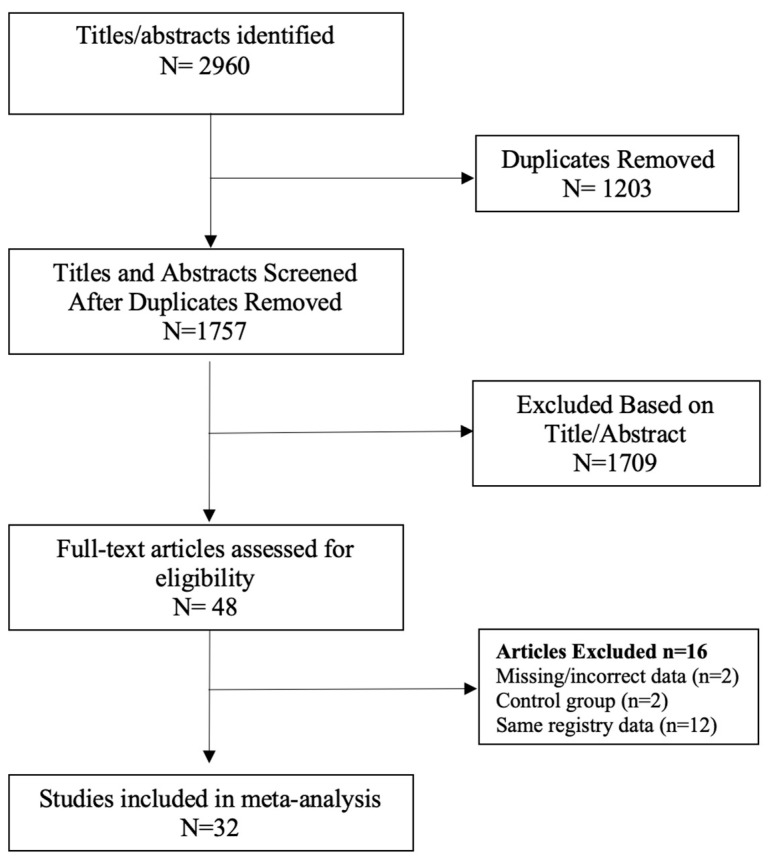
PRISMA flow chart [34,35].

The following limits were applied to all searches: English language, abstract included, human subjects and published in the last 15 years (January 2007–February 2023). Studies with a small sample size (less than 20 participants) were excluded. Duplicate search results were removed before screening for eligibility according to titles and abstracts. Systematic reviews and meta-analyses were excluded, as well as literature reviews, case reports, letters to the editor and narrative reviews. The full texts of the remaining studies were retrieved for further evaluation by two independent reviewers (DL and RBW). When multiple studies utilised the same registry data or patient population, the study with the largest sample size was included (Figure 1) [34,35]. Selection discrepancies were resolved through discussion and consensus by two independent reviewers (DL and RBW).

### 2.3. Risk of Bias Assessment

The risk of bias (RoB) was assessed using Cochrane’s ROBINS-I domain-based tool for observational studies with the assessment of confounding, selection of participants, classification of interventions, deviation from intended interventions, missing data, measurement of outcomes and selection of reported outcomes [36]. Studies were classified in their overall RoB outcome as low, moderate, serious or critical. Each RoB evaluation was discussed between the reviewers, and a third reviewer was consulted when required. RevMan Version 5.4.1 from the Cochrane Collaboration was used to display the results [36]. Publication bias was assessed by Egger’s test and funnel plot asymmetry for each meta-analysis and subgroup analysis using a random effects model and Microsoft Excel MetaXL 5.3 software. Funnel plots were plotted with standard error on the *y* axis versus log of the average effect (ln RR) on the *x* axis, and red dots represented each study in the respective meta-analysis for that group.

### 2.4. Data Extraction

Studies were referenced using Endnote 20 software, and Microsoft Excel was used during the screening and data extraction process. Data were extracted by two independent reviewers into an Excel spreadsheet for the study design, country of origin, type of cancer, procedure type, time period of study, duration of follow up, sample size (bariatric surgery vs. conventional treatment), gender, mean age of participants and preoperative BMI.

### 2.5. Statistical Analysis

Microsoft Excel and Cochrane Collaboration’s Revman (version 5.4.1) software were used to perform the statistical analysis. A meta-analysis of the dichotomous outcomes was conducted in Revman using the inverse variance method and the random effects model, as described by Der Simonian and Laird [36]. Each outcome required results from a minimum of three studies. The hazard ratio (HR), OR and relative risk (RR) were treated as equivalent measures of the risk, as they were all derived from cohort studies. The relative RR was calculated for each outcome, with a 95% confidence interval (95% CI). The percentage weight of each study was derived from the random effects analysis. The primary outcomes were the overall cancer incidence, obesity-associated cancer (OAC) incidence and cancer-related mortality. Subgroup analyses were conducted for the different types of OAC (breast cancer, endometrial cancer, ovarian cancer, colorectal cancer, oesophageal adenocarcinoma, hepatocellular carcinoma, kidney cancer, gall bladder cancer, multiple myeloma, pancreatic cancer, thyroid cancer and gastric cancer), as well as prostate cancer. Since most of the data were retrospective and derived from medical records or databases, the exact definition of OAC adopted by each study was noted. Forest plots were constructed using Revman (version 5.4.1), with the black diamond representing the pooled effect (RR) and the horizontal length of the diamond the confidence interval of the pooled result, with point estimates for each individual study plotted. Each point estimate was surrounded by a blue square, the size of which was determined by the weight of the effect size, and a horizontal bar the range of the confidence interval calculated for the observed effect size [37].

Heterogeneity was assessed using the I^2^ statistic and the Cochran’s Q statistical test. As per the Cochrane Online Handbook, I^2^ values of <30%, 30–60%, 50–90% and 75–100% respectively represented low, moderate, substantial and considerable heterogeneity [37]. A *p*-value < 0.05 was considered to demonstrate statistically significant heterogeneity across the studies. When substantial heterogeneity (I^2^ ≥ 50%) was present in a pooled meta-analysis, sensitivity analyses were performed to identify outliers. Outliers were removed when their 95% CIs were entirely outside the 95% CI of the pooled effect size. For all outcomes, influence analyses (‘leave one out’ analyses, i.e., recalculating the pooled effect size k times while omitting one study at a time) were performed to examine the influence of individual studies on the overall effect [35]. Tables were constructed for each influence analysis using Microsoft MetaXL 5.3 software. 

## 3. Results

A total of 2960 records were screened, and 47 studies were retrieved for further evaluation, as per the modified PRISMA flow diagram in Figure 1. Following application of the inclusion and exclusion criteria, 32 studies were included in the systematic review and meta-analysis [38,39,40,41,42,43,44,45,46,47,48,49,50,51,52,53,54,55,56,57,58,59,60,61,62,63,64,65,66,67,68,69]. There was a total of 511,585 patients in the bariatric surgery cohort compared to 1,889,746 control patients in the overall cancer risk analysis. Larger numbers of patients were available for analysis in specific cancer subtypes, including breast, colorectal cancer (CRC), hepatocellular carcinoma (HCC) and kidney cancer. The bariatric procedures performed included Roux-en-Y gastric bypass, sleeve gastrectomy, gastric banding (LAGB), vertical banded gastroplasty (VBG), duodenal switch/BPD and other/unknown. The further breakdown of specific bariatric procedures, study characteristics and patient demographic details is summarised in Table 1. Of the 25/32 studies in which male and female data were reported, the majority of patients undergoing bariatric surgery were female (77.6%). There were three prospective, non-randomised, cross-sectional, matched cohort surgical intervention studies included, all from the Swedish Obesity Subjects (SOS) trial [48,53,59]. The remaining studies were either national population, health provider, provincial or registry-based retrospective, matched cohort studies. The studies were based mainly in North America and Europe, including the USA; the UK; Canada; Sweden; Denmark; Nordic group (Denmark, Finland, Iceland, Norway and Sweden); France, Italy and Taiwan. Forest plots depicting the pooled random effects analyses for each group/cancer are shown in Meta-Analysis Results (Random Effects Analysis).

### Risk of Bias (RoB)

**Figure 2:**
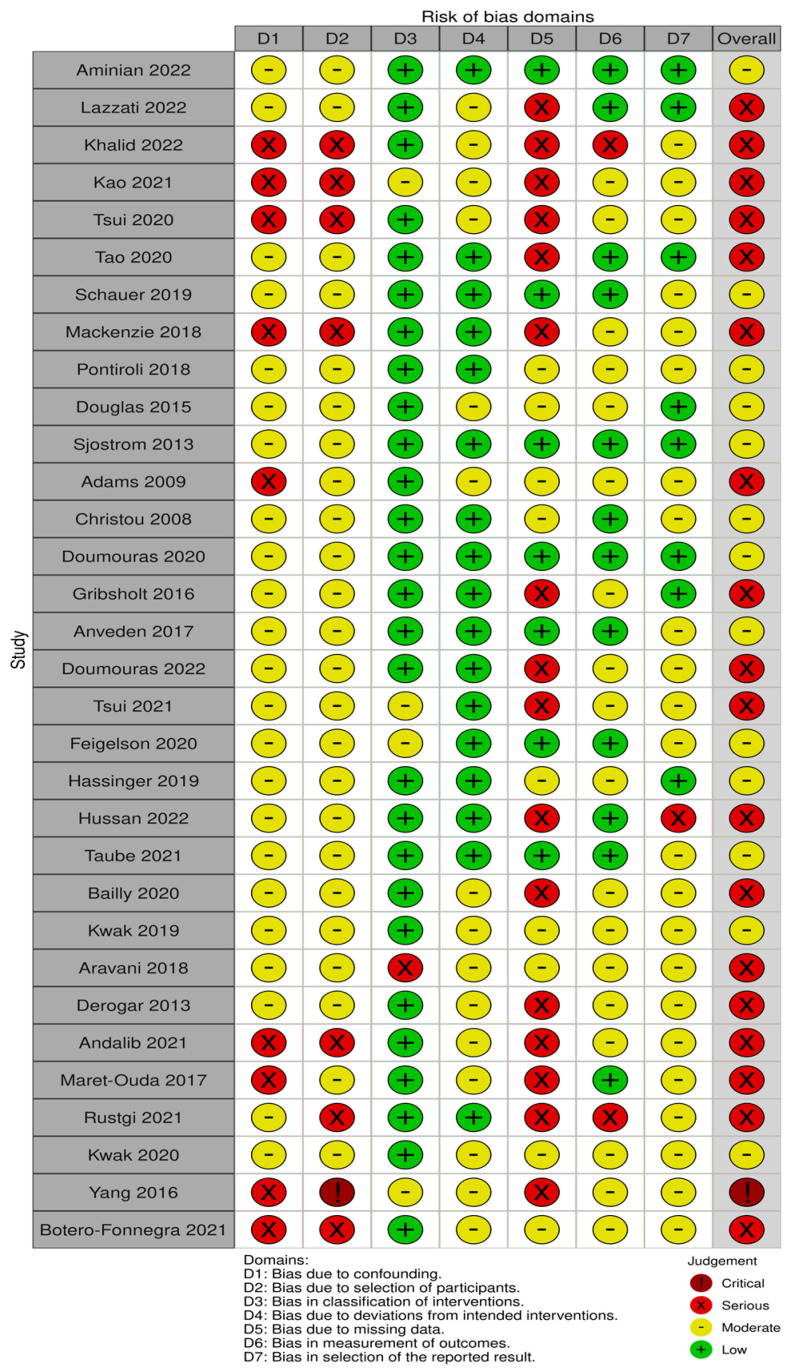
Risk of bias assessment in 32 studies based on 7 domains [38,39,40,41,42,43,44,45,46,47,48,49,50,51,52,53,54,55,56,57,58,59,60,61,62,63,64,65,66,67,68,69].

**Figure A1:**
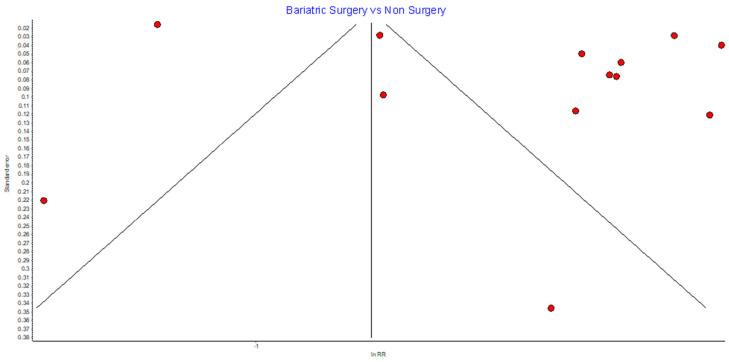
Overall cancer incidence RE model funnel plot.

**Figure A2:**
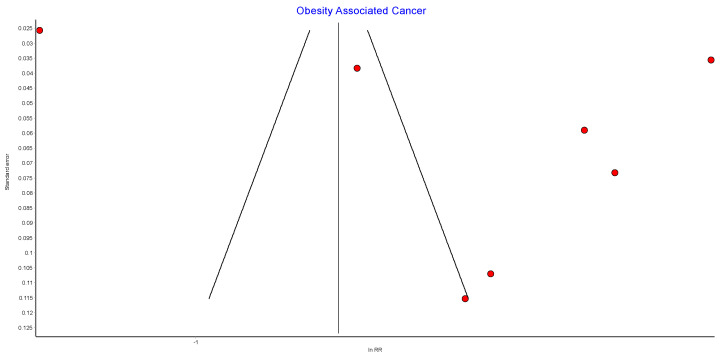
Incidence of obesity-associated cancers (OAC) RE model funnel plot.

**Figure A3:**
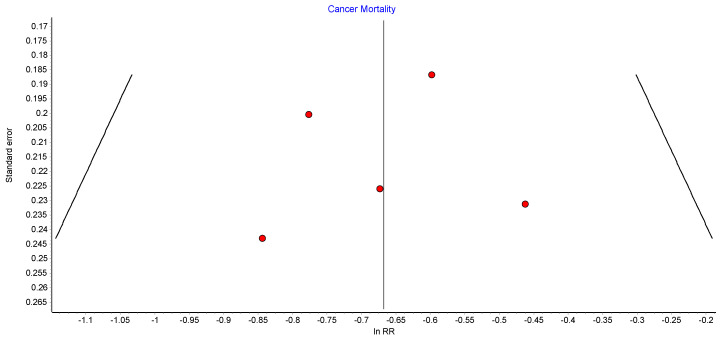
Cancer-related mortality RE funnel plot.

**Figure A4:**
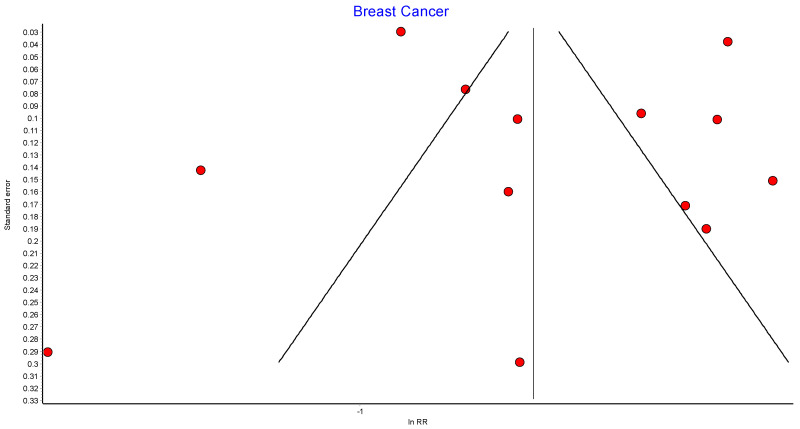
Overall breast cancer random effects model funnel plot.

**Figure A5:**
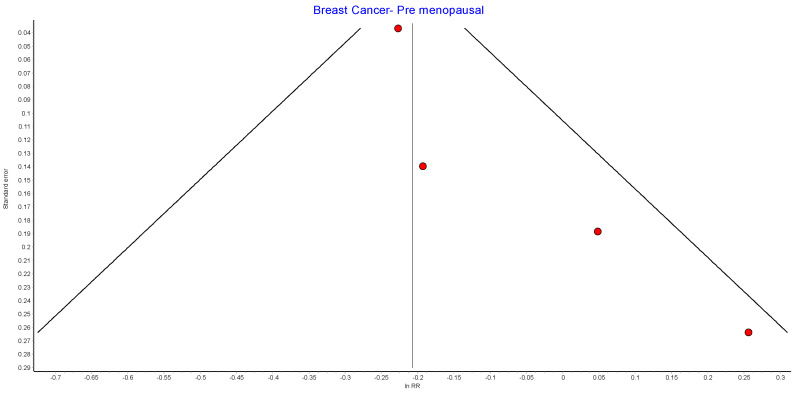
Premenopausal breast cancer random effects model funnel plot.

**Figure A6:**
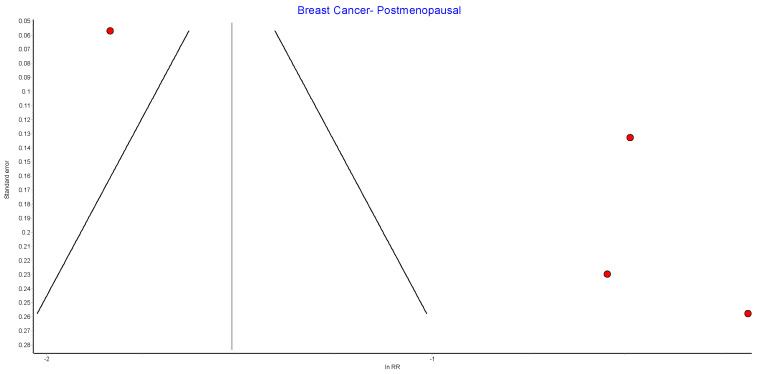
Postmenopausal breast cancer random effects model funnel plot.

**Figure A7:**
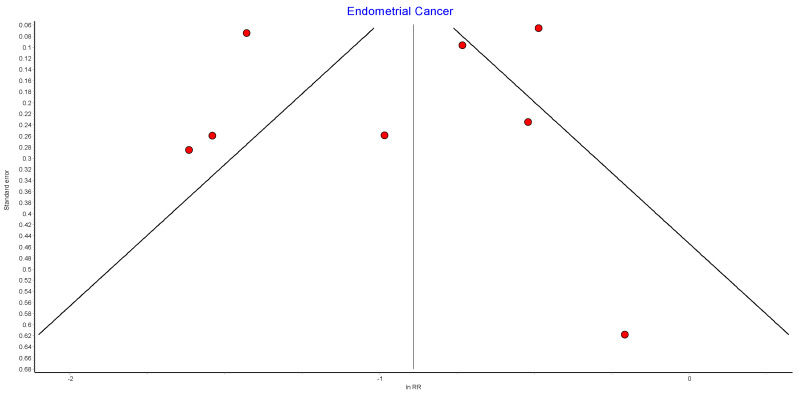
Endometrial cancer random effects model.

**Figure A8:**
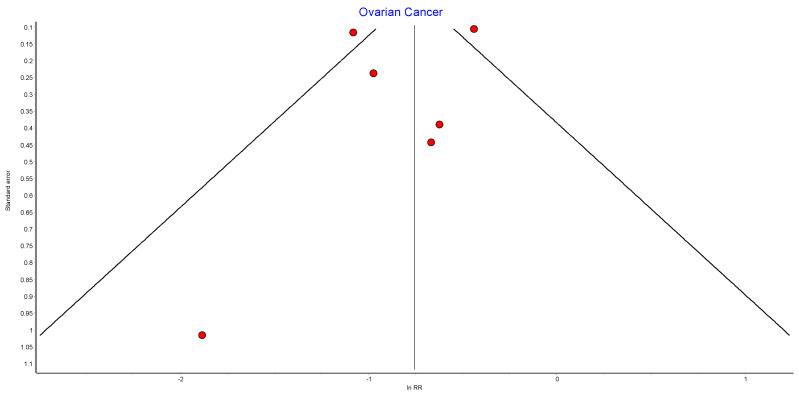
Ovarian cancer random effects model funnel plot.

**Figure A9:**
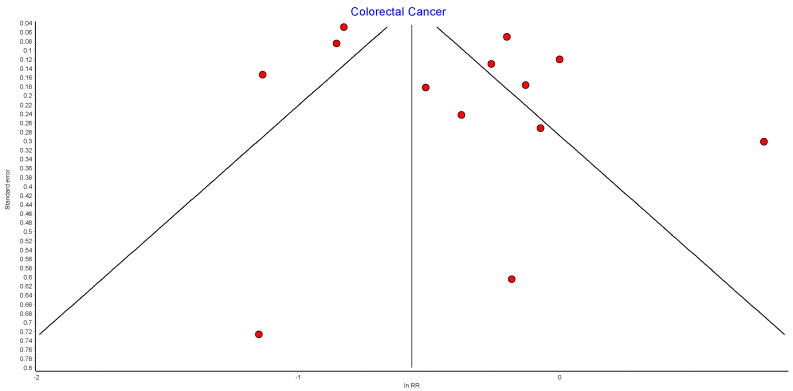
Colorectal cancer random effects model funnel plot.

**Figure A10:**
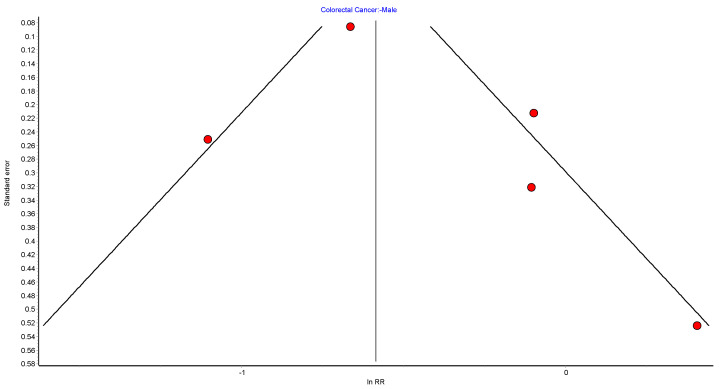
CRC male random effects model funnel plot.

**Figure A11:**
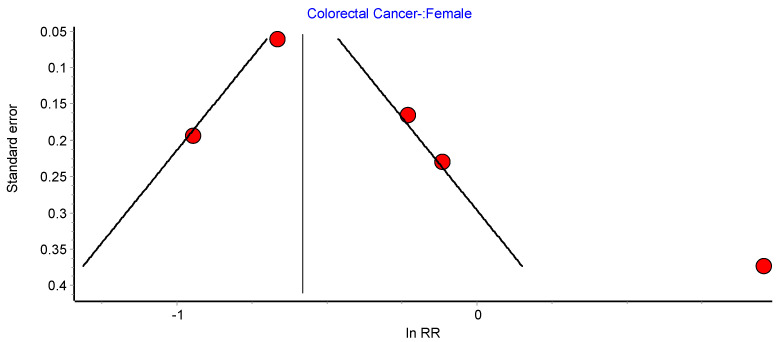
CRC female random effects model funnel plot.

**Figure A12:**
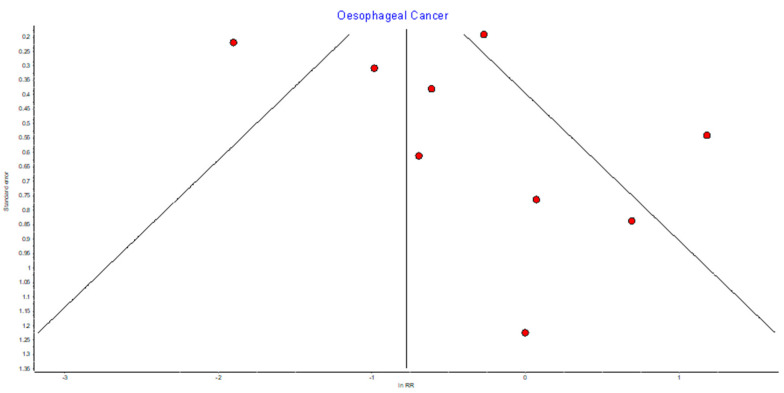
Oesophageal random effects model funnel plot.

**Figure A13:**
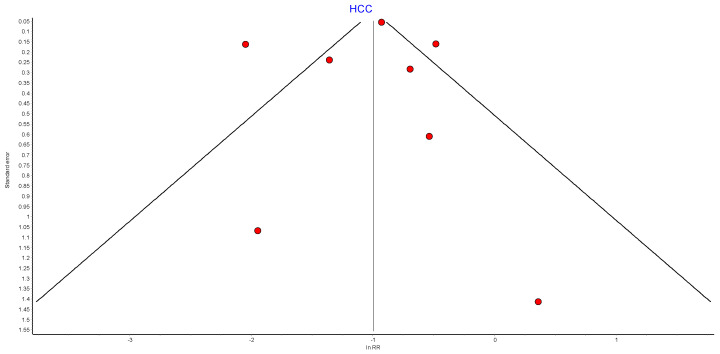
HCC RE model funnel plot.

**Figure A14:**
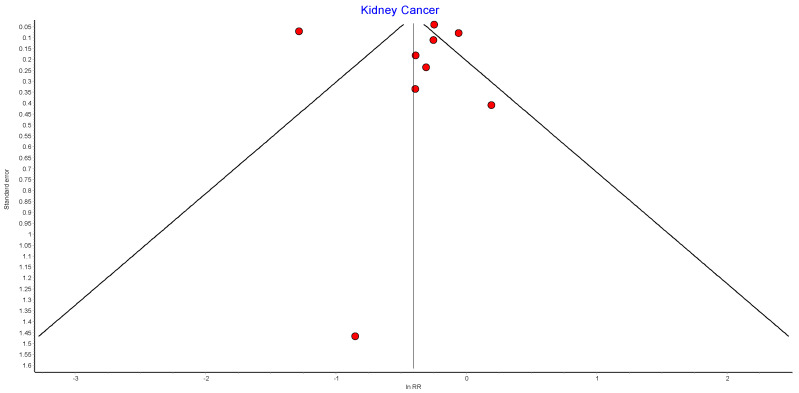
Kidney cancer random effects model funnel plot.

**Figure A15:**
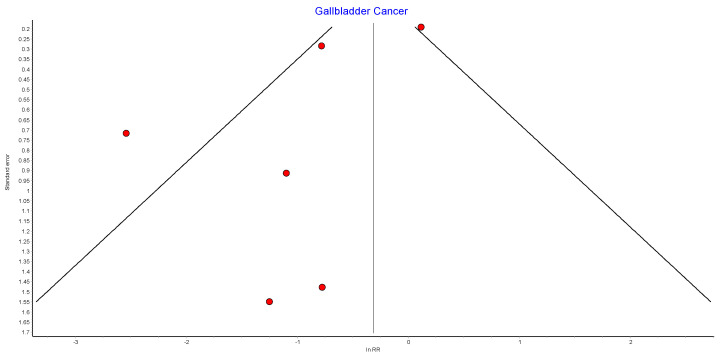
Gallbladder cancer random effects model funnel plot.

**Figure A16:**
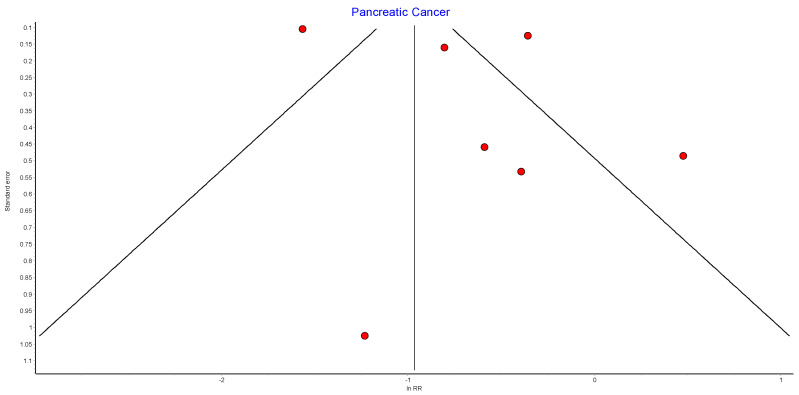
Pancreatic cancer random effects model funnel plot.

**Figure A17:**
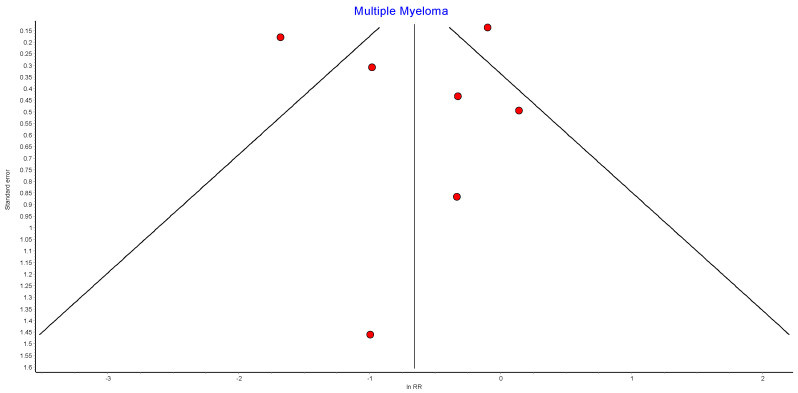
Multiple myeloma random effects model funnel plot.

**Figure A18:**
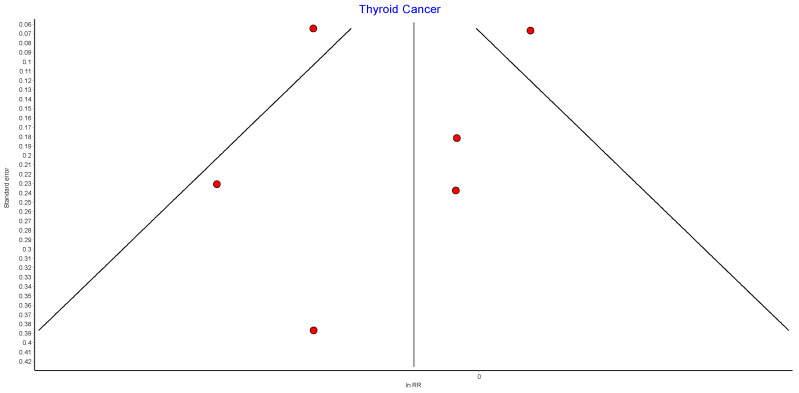
Thyroid cancer random effects model funnel plot.

**Figure A19:**
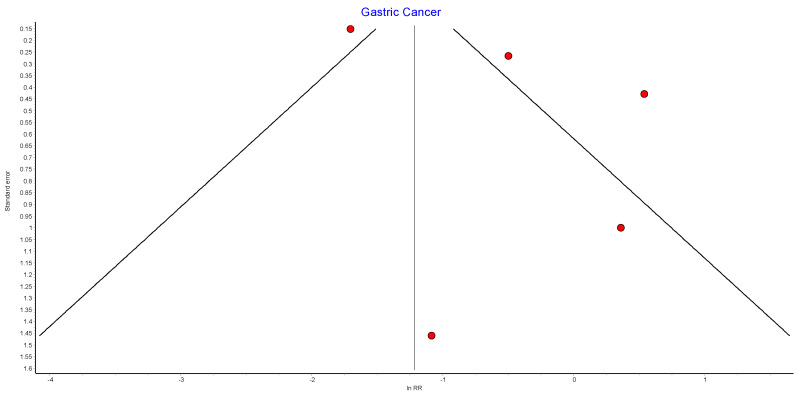
Gastric cancer random effects model funnel plot.

**Figure A20:**
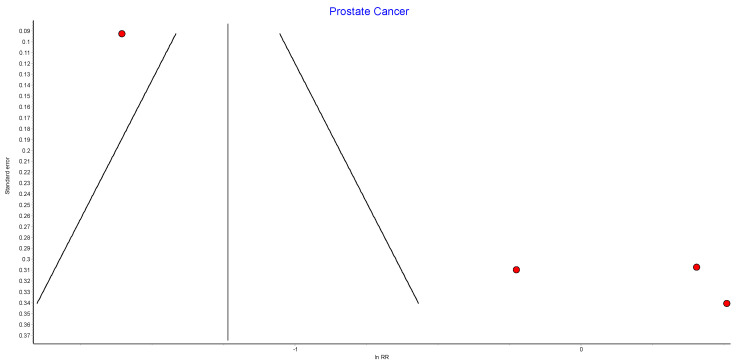
Prostate cancer random effects model funnel plot.

Thirteen out of thirty-two studies had a moderate risk of bias [38,44,46,47,48,50,51,53,56,57,59,61,67], eighteen studies had a serious risk of bias [39,40,41,42,43,45,49,52,54,55,58,60,62,63,64,65,66,69] and one critical RoB [68] (Figure 2). The main sources of bias consisted of confounding bias, selection bias and missing data due to the observational nature of the studies included in this SRMA. A publication bias analysis was performed and represented in Funnel plots in Figure A1, Figure A2, Figure A3, Figure A4, Figure A5, Figure A6, Figure A7, Figure A8, Figure A9, Figure A10, Figure A11, Figure A12, Figure A13, Figure A14, Figure A15, Figure A16, Figure A17, Figure A18, Figure A19 and Figure A20. Asymmetry was found in the funnel plots for overall cancer studies, breast and colorectal cancer. These were the only groups that contained more than 10 studies. Other reasons for funnel plot asymmetry apart from publication bias include high heterogeneity, inclusion of large and small studies with clinical or methodological differences or choice of statistical analysis [37].

A sensitivity analysis is represented in the ‘leave one out’ analyses in Table A1, Table A2, Table A3, Table A4, Table A5, Table A6, Table A7, Table A8, Table A9, Table A10, Table A11, Table A12, Table A13, Table A14, Table A15, Table A16, Table A17, Table A18, Table A19 and Table A20. The study from Mackenzie et al. [45] was identified as a significant outlier in the analysis of incidence of colorectal cancer after bariatric surgery, leading to re-analysis of the overall, male and female CRC pooled effects. The studies from Lazzati et al. [39] and Adams et al. [49] appeared to influence the subset analysis for the pre- and postmenopausal breast cancer results, presumably due to the large differences in cohort size and the small number of studies with sufficient data. The study from Lazzati et al. [39] was also identified to have a size effect in the sensitivity analysis on the cancer incidence of RR in gastric, pancreatic, gall bladder, thyroid, kidney, prostate cancer and multiple myeloma, and also contributed to the significant heterogeneity between studies (Appendix A).

## 4. Meta-Analysis Results (Random Effects Analysis)

### 4.1. Overall Cancer Incidence

**Figure 3:**
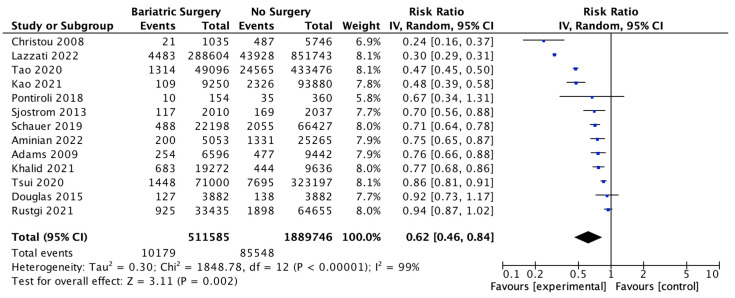
Overall cancer incidence RE analysis [38,39,40,41,42,43,44,46,47,48,49,50,66].

Thirteen studies reported the overall incidence of cancer with 511,585 patients in the bariatric surgery cohort compared to 1,889,746 in the control group. Bariatric surgery was associated with a significant reduction in the overall incidence of cancer, which includes both OAC and cancers that have been previously classified as ‘non-obesity-associated cancers’ (RR 0.62, 95% CI 0.46–0.84, *p* = 0.002). There was statistically significant heterogeneity across the studies (Chi^2^ = 1848.78, *p* < 0.00001, I^2^ = 99%) (Figure 3).

### 4.2. Incidence of Obesity-Associated Cancers (OAC)

**Figure 4:**
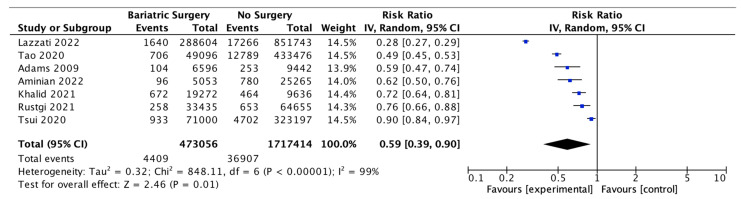
Incidence of obesity-associated cancers (OAC) [38,39,40,42,43,49,66].

Seven studies reported the incidence of obesity-associated cancer (OAC), with 473,056 patients in the bariatric surgery cohort compared to 1,717,414 in the control group. All studies used the 2016 IARC/US CDC classification of 13 OACs, except for Adams et al. 2009 [48], who included oesophageal adenocarcinoma, colorectal, pancreas, postmenopausal breast, uterine, kidney, liver and gallbladder cancer; non-Hodgkin’s lymphoma; leukaemia and multiple myeloma and excluded ovarian, gastric and thyroid cancer based on earlier classifications. In our meta-analysis, bariatric surgery was associated with a significant reduction in the incidence of OAC (RR 0.59, 95% CI 0.39–0.90, *p* = 0.01). There was statistically significant heterogeneity across the studies (Chi^2^= 848.11, *p* < 0.00001, I^2^ = 99%) (Figure 4).

### 4.3. Cancer-Related Mortality

**Figure 5:**
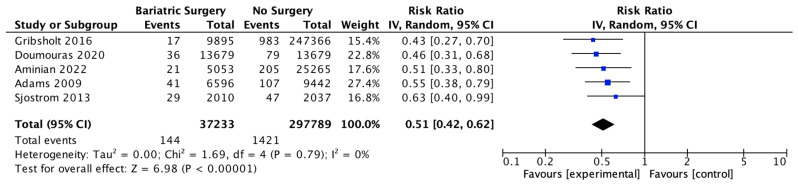
Cancer-related mortality [38,48,49,51,52].

Five studies reported on the incidence of cancer-related mortality, with 37,233 patients in the bariatric surgery cohort compared to 297,789 in the control group. Bariatric surgery was associated with a significant reduction in the incidence of cancer-related mortality (RR 0.51, 95% CI 0.42–0.62, *p* < 0.00001). There was no heterogeneity observed across the studies (Chi^2^ = 1.69, *p* = 0.79, I^2^ = 0) (Figure 5).

### 4.4. Breast Cancer

Thirteen studies reported the incidence of breast cancer, with 425,846 female patients in the bariatric surgery cohort compared to 1,757,986 female patients in the control group. Bariatric surgery was associated with a significant reduction in the overall risk of developing breast cancer (RR 0.56, 95% CI 0.44–0.71, *p* < 0.00001). There was statistically significant heterogeneity across the studies (Chi^2^= 340.97, *p* < 0.00001, I^2^ = 96%).

**Figure 6:**
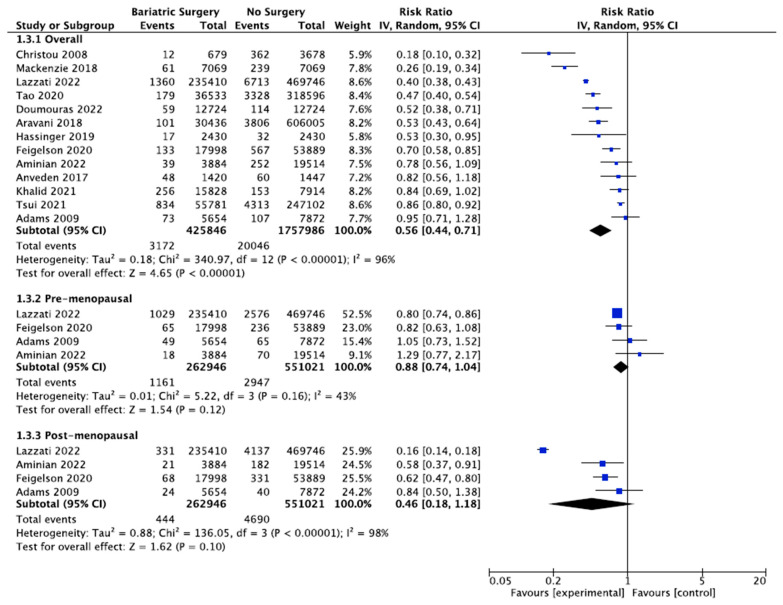
Breast cancer incidence [38,39,40,43,45,49,50,53,54,55,56,57,62].

Further subgroup analyses were performed using data from four of the studies that examined the incidence of both premenopausal and postmenopausal breast cancer in 262,946 patients who received bariatric surgery and 551,021 control patients [38,39,49,56]. There was a nonsignificant reduced incidence of postmenopausal breast cancer (RR 0.46, 95% CI 0.18–1.18, *p* = 0.10) and no significant risk reduction for premenopausal breast cancer (RR 0.88, 95% CI 0.74–1.04, *p* = 0.12) in obese women who received bariatric surgery. There was moderate heterogeneity for the premenopausal breast cancer analysis (Chi^2^= 5.22, *p* = 0.16, I^2^ = 43%), and high heterogeneity for the postmenopausal breast cancer analysis (Chi^2^= 136.05, *p* = 0.00001, I^2^ = 98%) (Figure 6).

### 4.5. Endometrial Cancer

**Figure 7:**
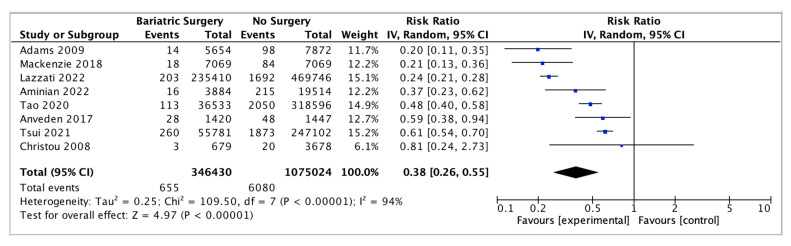
Endometrial cancer incidence [38,39,43,45,49,50,53,55].

Eight studies reported on endometrial cancer incidence, with 346,430 female patients in the bariatric surgery cohort compared to 1,075,024 female patients in the control group. Bariatric surgery was associated with a significant reduction in the incidence of endometrial cancer (RR 0.38, 95% CI 0.26–0.55, *p* < 0.00001). There was statistically significant heterogeneity across the studies (Chi^2^ = 109.50, *p* <0.00001, I^2^ = 94%) (Figure 7).

### 4.6. Ovarian Cancer

**Figure 8:**
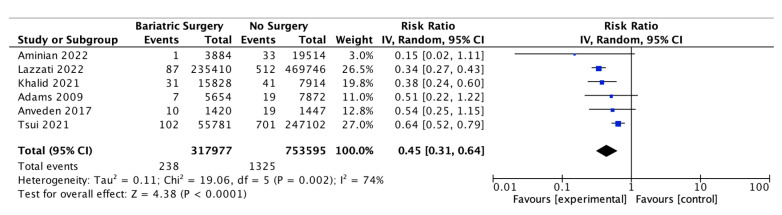
Ovarian cancer incidence [38,39,40,49,53,55].

Six of the studies reported on the incidence of ovarian cancer, with 317,977 female patients in the bariatric surgery cohort compared to 753,595 female patients in the control group. Bariatric surgery was associated with a significant decrease in the incidence of ovarian cancer (RR 0.45, 95% CI 0.31–0.64, *p* < 0.0001). There was significant heterogeneity between the studies (Chi^2^ = 19.06, *p* = 0.002, I^2^ = 74%) (Figure 8).

### 4.7. Colorectal Cancer

Thirteen studies reported on the incidence of colorectal cancer, with 382,686 patients in the bariatric surgery cohort compared to 3,143,652 in the control group. Bariatric surgery was associated with a significant reduction in the incidence of colorectal cancer (RR 0.69, 95% CI 0.53–0.88, *p* = 0.003). There was statistically significant heterogeneity across the studies (Chi^2^ = 138.71, *p* < 0.00001, I^2^ = 91%).

Subgroup analyses were conducted across five studies to compare the incidence of CRC in males and females after bariatric surgery. There was a total of 31,441 males who received bariatric surgery and 873,571 in the control group. The sample size for female participants was greater, with 108,332 in the surgery group and 1,133,354 controls. There was a greater risk reduction for the male subgroup (RR 0.65, 95% CI 0.43–0.96, *p* = 0.03) than the female subgroup (RR 0.75, 95% CI 0.49–1.13, *p* = 0.17) who underwent bariatric surgery compared to those with conventional treatment for obesity.

**Figure 9:**
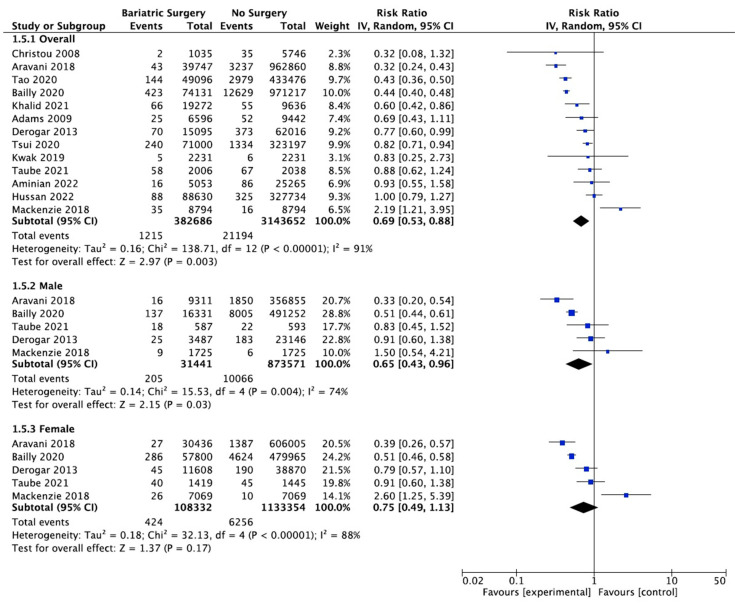
Colorectal cancer incidence [38,40,42,43,45,49,50,58,59,60,61,62,63].

**Figure A21:**
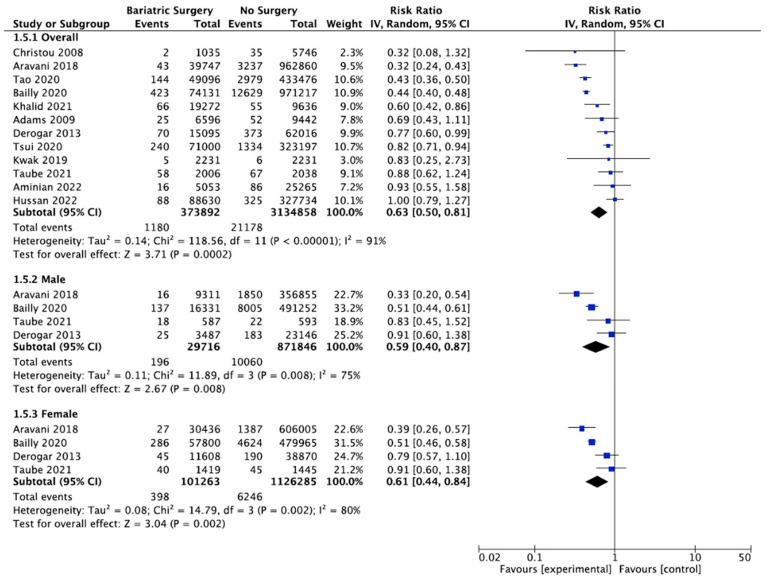
Future CRC risk incidence. Forest plot with Mackenzie et al. [45] data removed from the overall and subgroup CRC analysis after the sensitivity analysis [38,40,42,43,49,50,58,59,60,61,62,63].

However, in the sensitivity analysis (Table A9), with removal of the study by Mackenzie et al. (2018), the results were substantially different [45]. There was an overall significant CRC risk reduction with bariatric surgery (RR 0.63, CI 0.50–0.81, *p* = 0.0002) but with persistent significant heterogeneity (Chi^2^ = 118.56, *p* < 0.00001, I^2^ = 91%). The risk reduction was now similar between males (RR 0.59, 95% CI 0.40–0.87, *p* = 0.008) and females (RR 0.61, 95% CI 0.44–0.84, *p* = 0.002). There was considerable heterogeneity between the five studies for both males (Chi^2^ = 15.53, *p* = 0.004, I^2^ = 74%) and females (Chi^2^ = 32.13, *p* < 0.00001, I^2^ = 88%) (Figure 9). This remained in the four pooled studies for both male data (Chi^2^ = 11.89, *p* = 0.008, I^2^ = 75%) and female data (Chi^2^ = 14.79, *p* = 0.002, I^2^ = 80%) after the study by Mackenzie et al. [45] was excluded (Figure A21).

### 4.8. Oesophageal Adenocarcinoma

**Figure 10:**
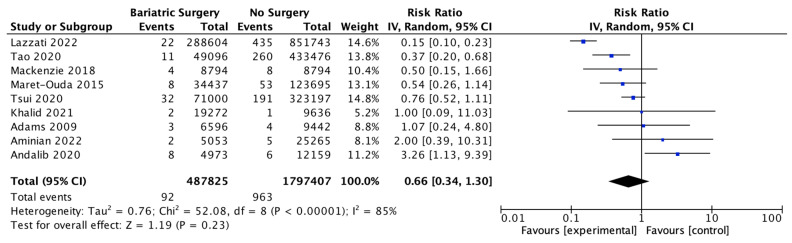
Oesophageal adenocarcinoma incidence [38,39,40,42,43,45,49,64,65].

Nine studies investigated the incidence of oesophageal adenocarcinoma, with 487,825 patients in the bariatric surgery cohort compared to 1,797,407 in the control group. Bariatric surgery was associated with a nonsignificant reduction in the incidence of oesophageal adenocarcinoma (RR 0.66, 95% CI 0.34–1.30, *p* = 0.23). There was significant heterogeneity between the studies (Chi^2^ = 52.08, *p* < 0.00001, I^2^ = 85%) (Figure 10).

### 4.9. Hepatocellular Carcinoma

**Figure 11:**
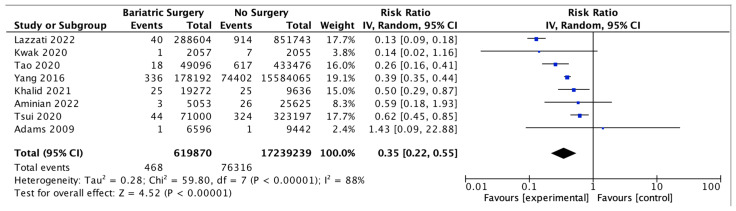
Hepatocellular carcinoma incidence [38,39,40,42,43,49,67,68].

Eight studies assessed the impact of bariatric surgery on HCC, with 619,870 patients in the bariatric surgery cohort compared to 17,239,239 in the control group. Bariatric surgery was associated with a significant reduction in the incidence of HCC (RR 0.35, 95% CI 0.22–0.55, *p* < 0.00001). There was significant heterogeneity across the studies (Chi^2^ = 59.80, *p* < 0.00001, I^2^ = 88%) (Figure 11).

### 4.10. Kidney Cancer

**Figure 12:**
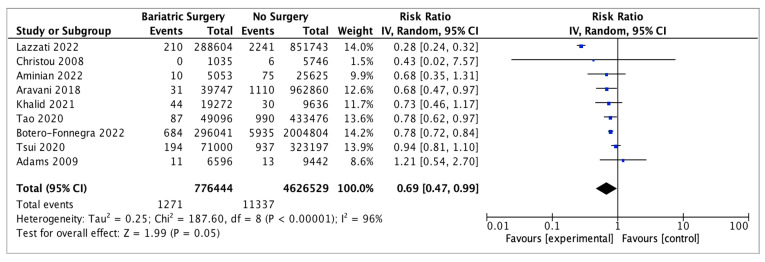
Kidney cancer incidence [38,39,40,42,43,49,50,62,69].

Nine studies reported on kidney cancer incidence, with 776,444 patients in the bariatric surgery cohort compared to 4,626,529 in the control group. Bariatric surgery was associated with a nonsignificant reduction in the risk of kidney cancer (RR 0.69, 95% CI 0.47–0.99, *p* = 0.05). There was significant heterogeneity across the studies (Chi^2^ = 187.60, *p* < 0.00001, I^2^ = 96%) (Figure 12).

### 4.11. Pancreatic Cancer

**Figure 13:**
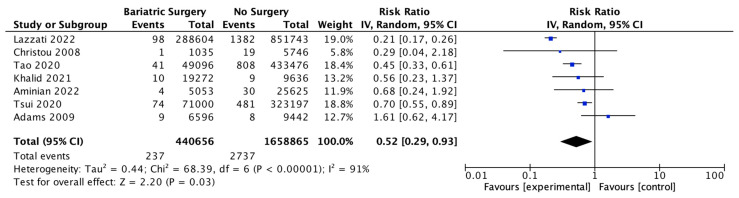
Pancreatic cancer incidence [38,39,40,42,43,49,50].

Seven studies reported pancreatic cancer incidence, with 440,656 patients in the bariatric surgery cohort compared to 1,658,865 in the control group. Bariatric surgery was associated with a significant reduction in the risk of developing pancreatic cancer (RR 0.52, 95% CI 0.29–0.93, *p* = 0.03). There was significant heterogeneity across the studies (Chi^2^ = 68.39, *p* < 0.00001, I^2^ = 91%) (Figure 13).

### 4.12. Gallbladder Cancer

**Figure 14:**
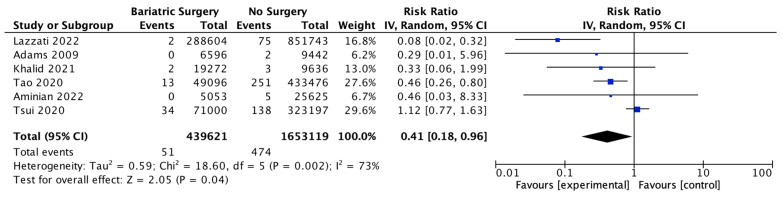
Gallbladder cancer incidence [38,39,40,42,43,49].

Six studies reported on gal1bladder cancer, with 439,621 patients in the bariatric surgery cohort compared to 1,653,119 in the control group. Bariatric surgery was associated with a significant reduction in the incidence of gallbladder cancer (RR 0.41, 95% CI 0.18–0.96, *p* = 0.04). There was statistically significant heterogeneity across the studies (Chi^2^ = 18.60, *p* = 0.002, I^2^ = 73%) (Figure 14).

### 4.13. Multiple Myeloma

**Figure 15:**
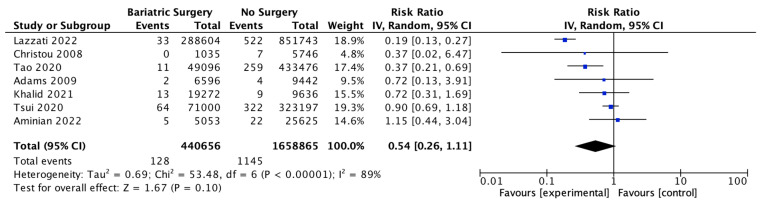
Multiple myeloma incidence [38,39,40,42,43,49,50].

Seven studies reported outcomes for multiple myeloma, with 440,656 patients in the bariatric surgery cohort compared to 1,658,865 in the control group. There was a trend to risk reduction in the incidence of multiple myeloma (RR 0.54, 95% CI 0.26–1.11, *p* = 0.10). There was significant heterogeneity across the studies (Chi^2^ = 53.48, *p* < 0.00001, I^2^ = 89%) (Figure 15).

### 4.14. Thyroid Cancer

**Figure 16:**
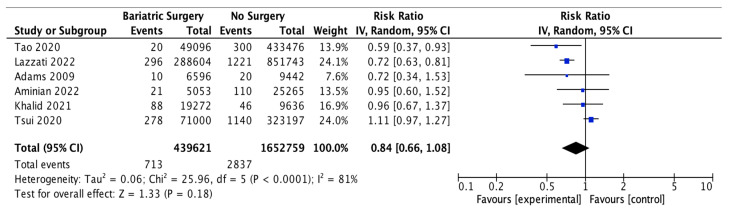
Thyroid cancer incidence [38,39,40,42,43,49].

Six studies reported outcomes for thyroid cancer incidence, with 439,621 patients in the bariatric surgery cohort compared to 1,652,759 in the control group. Bariatric surgery was associated with a nonsignificant reduction in the incidence of thyroid cancer (RR 0.84, 95% CI 0.66–1.08, *p* = 0.18). There was statistically significant heterogeneity between the studies (Chi^2^ = 25.96, *p* < 0.0001, I^2^ = 81%) (Figure 16).

### 4.15. Gastric Cancer

**Figure 17:**
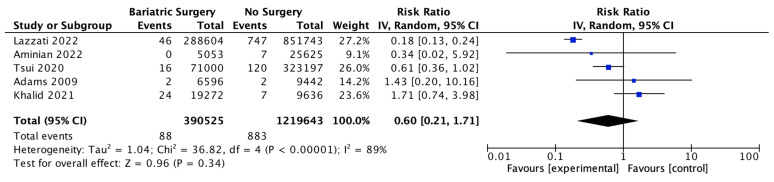
Gastric cancer incidence [38,39,40,42,49].

Five studies reported on gastric cancer incidence, with 390,525 patients in the bariatric surgery cohort compared to 1,219,643 patients in the control group. There was a nonsignificant reduction in the risk of gastric cancer after bariatric surgery (RR 0.60, 95% CI 0.21–1.71, *p* = 0.34). There was significant heterogeneity between the studies (Chi^2^= 36.82, *p* = 0.00001, I^2^ = 89%) (Figure 17).

### 4.16. Prostate Cancer

**Figure 18:**
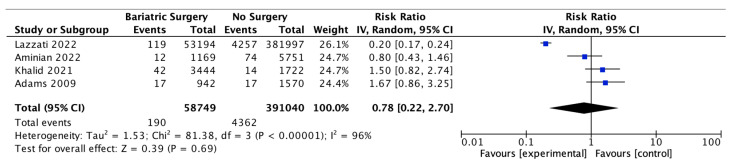
Prostate cancer incidence [38,39,40,49].

Four studies reported on prostate cancer incidence, with 58,749 male patients in the bariatric surgery cohort compared to 391,040 males in the control group. There was no significant difference in prostate cancer incidence between bariatric surgery and no surgery groups (RR 0.78, 95% CI 0.22–2.70, *p* = 0.69). There was significant heterogeneity between the studies (Chi^2^ = 81.38, *p* = 0.00001, I^2^ = 96%) (Figure 18).

## 5. Discussion

### 5.1. Metabolic Syndrome and Cancer Risk following Bariatric Surgery

When compared to conventional treatment, BMS results in more substantial and durable weight loss while improving inflammatory markers, insulin sensitivity (HOMA-IR) and resolving other underlying disease processes, including metabolic syndrome, T2DM, glycosylated haemoglobin (HbA1c), NASH, aromatase activity, visceral obesity, hypoxia and OSA [23,31,69,70,71,72,73,74,75,76,77,78,79,80,81,82,83,84,85,86,87,88,89,90,91,92,93,94,95,96,97,98]. RYGB surgery has been shown to decrease macrophage infiltration and crown-like structures (CLS) in subcutaneous adipose tissue (SAT), indicating improvement in local white adipose tissue (WAT) inflammation. This may also relate to improvements in WAT senescence-associated secretory phenotype (SASP) and adipokine release [70]. For example, both LSG and RYGB are associated with rapid and sustained decrease in fasting serum leptin levels (males: 31.3 ± 10.5 vs. 6.1 ± 5.5 ng/mL, *p* < 0.001; females: 60.2 ± 14.3 vs. 12.5 ± 9.4 ng/mL, *p* < 0.001) and doubling in fasting serum adiponectin levels (males: 3.4 ± 1.6 vs. 8.9 ± 4.8 ng/mL; females 6.1 ± 1.8 vs. 11.6 ± 3.7 ng/mL, *p* < 0.001), respectively, comparing preoperative and postoperative levels 12 months after surgery. The greatest improvements in adipokines after BMS occur in patients with higher preoperative BMI and metabolic syndrome [23,31,97,99,100]. The decrease in leptin, cytokines, PGE2 and NF-κB activation and the resultant lowering of WAT aromatase activity after bariatric surgery are associated with larger postoperative falls in the serum oestradiol at 12 months after surgery in older women (53.9 to 35.7 pg/mL) as compared to oestradiol in younger women (94.85 to 73.62 pg/mL). Changes in oestrogen and testosterone metabolism and the LH, FSH and SHBG levels after bariatric surgery result in the improvement of polycystic ovary syndrome (96% resolution) and fertility in premenopausal women, a potential decrease in future oestrogen-sensitive cancers in women and hypogonadism (87% resolution) in males [72,89]. 

Aminian et al. (2022) demonstrated increasing divergence after six years of follow-up of their Kaplan–Meier curves for obesity-associated cancer (OAC) incidence when comparing bariatric surgery and conventional weight loss interventions [38]. This suggests that significant and long-term weight loss (together with the resolution of metabolic syndrome) is required to mitigate the future risk of developing cancer [38]. Schauer et al. (2017) demonstrated improvements in the overall cancer-free survival in patients 10 years after BMS [101]. The greatest cancer-free survival was experienced by individuals who lost >30% of their TBW at one year post-BMS as compared to individuals who lost 20–30%, with the worst outcomes in individuals who lost <20% of their TBW. The average TBW loss after BMS at 1 year was 27% compared to 1% in the control group. Each 10% loss of the TBW at one year post-BMS was associated with a 14% lower risk of future cancer development [101]. 

There is evidence to suggest that, although exercise, a Mediterranean-style diet and medical management may not be successful in patients with Class II and III obesity, they can be synergistic when combined with bariatric surgery in reversing metabolic syndrome, maintaining a robust postoperative weight loss and potentially contributing to a decrease in future cancer risk [25,97]. This is likely related to continuing adipokine and exosome release from dysfunctional adipocytes and abnormal neurohormonal homeostasis if patients resume a poor diet or sedentary lifestyle with associated weight regain post-surgery. The observation that plasma extracellular vesicles (80% microvesicles, 100–1000 nm; 20% exosomes, 20–100 nm diameter) are increased tenfold in number in obese patients and do not return to normal even after sustained weight loss from caloric restriction, diet plus exercise or LSG suggests a permanent alteration of the adipocyte secretome [74,102]. However, alterations in the cargo of adipocyte-derived exosomes after successful BMS include a decline in FABP-4 and changes in 10 miRNAs that are involved in improved insulin signalling and lipid metabolism. Such changes are associated with greatly reduced markers of systemic inflammation, including serum C-reactive protein (CRP), TNF-α and IL-6 [71,73].

It should be noted that up to one in six patients will regain >10% of their total body weight after BMS (RYGB and LSG). This is relevant to the ‘dose-related’ effect of BMS on the cancer risk and metabolic syndrome and the design and capture of data of comparative studies. The risk factors for weight regain are categorised as temporal, dietary, neuropsychiatric, anatomical and genetic. Specific risk factors for postoperative weight regain include time after BMS; low physical activity; impulsive, disinhibited or maladaptive eating behaviour; low protein/highly refined carbohydrate dense meals; poor patient compliance; limbic/hypothalamic μ-opiate receptor (MOR) phenotype and food reward; larger gastric volume following sleeve gastrectomy (252.7 mL vs. 148.5 mL) and the gastro-jejunal stomal diameter after RYGB. Predictors of sustained weight loss after BMS include postoperative HDL levels, fruit and zinc consumption, postprandial GLP-1 levels, patient self-esteem, social support and willingness to engage in physical activity [103,104].

### 5.2. Summary of SRMA Results

The current systematic review and meta-analysis demonstrated a reduction in the overall incidence of cancer (RR 0.62, 95% CI 0.46–0.84, *p* < 0.002) and OAC (RR 0.59, 95% CI 0.39–0.90, *p* = 0.01) as well as reduced overall cancer-associated mortality (RR 0.51, 95% CI 0.42–0.62, *p* < 0.00001), in patients who received bariatric surgery. Previous studies have reported similar findings, which are related to the diverse and synergistic endocrine, metabolic and molecular changes following bariatric surgery [105,106,107,108]. There have been a number of large cohort studies published since 2020 that have contributed to the available evidence for a potential reduction in future cancers after BMS, including those with less frequent events. There are also controversies that can be explored with additional data, such as the risk of future cardio-oesophageal or colorectal cancer after BMS and the possible protective effect of obesity on the development of premenopausal breast cancer.

Our study provides a pooled analysis of the most recently published data of the effects of bariatric surgery on future cancer incidence, including cancers that are obesity-associated or sex hormone-sensitive and cancers that are not classified as such. In our subgroup meta-analysis, seven OACs (breast, endometrial, ovarian, hepatocellular, pancreatic, gallbladder and colorectal cancer) had significant reductions in incidence after bariatric surgery, but this risk reduction was not demonstrated for renal, thyroid, prostate, oesophageal or gastric cancers or for multiple myeloma.

#### 5.2.1. Oestrogen-Sensitive Cancers

The systematic review found a lower incidence of hormone-sensitive and female-specific cancers, including overall breast cancer (RR 0.56, 95% CI 0.44–0.71, *p* < 0.00001), endometrial cancer (RR 0.38, 95% CI 0.26–0.55, *p* < 0.00001) and ovarian cancer (RR 0.45, 95% CI 0.31–0.64, *p* < 0.0001) after bariatric surgery. A further subgroup analysis involving breast cancer incidence was performed using data from four of the studies. A nonsignificant risk reduction was found in an incidence of postmenopausal breast cancer (0.46, 95% CI 0.18–1.18, *p* = 0.10) and no significant risk reduction for premenopausal breast cancer incidence (RR 0.88, 95% CI 0.74–1.04, *p* = 0.12) in patients who received bariatric surgery [38,39,49,56]. There was also a nonsignificant reduction in the incidence of thyroid cancer (RR 0.84, 95% CI 0.66–1.08, *p* = 0.18), which shares similar risk factors, including female sex, obesity and ER-α stimulation by oestrogen [2]. The reduction in oestrogen-sensitive cancers is consistent with other systematic reviews [105,107,108] and is most likely related to the reduction in aromatase activity, plasma and tissue oestrogen and serum leptin and insulin following bariatric surgery [109,110]. This correlates with Anveden et al. (2017) and Sjöholm et al. (2021), who reported that women with baseline hyperinsulinemia experienced a greater reduction in the risk of female-specific cancers after bariatric surgery in the SOS trial [53,109]. Obesity results in cross-talks between leptin, ER-α receptor, IGF signalling and the transactivation of EGFR, which is thought to increase the risk of postmenopausal breast cancer. Other studies have suggested obesity *decreases* the risk of developing premenopausal breast cancer [74,83,110]. The influence of the ER status in obesity-related breast cancer was elucidated by Feigelson et al. (2020), who found that bariatric surgery significantly reduced the future risk of ER+ postmenopausal breast cancer (HR 0.51, 95% CI, 0.38–0.69, *p* <0.001) but not ER- postmenopausal breast cancer (HR 0.79, 95% CI, 0.41–1.51, *p* = 0.47) [56]. Conversely, bariatric surgery was protective for the future risk of premenopausal ER- breast cancer (HR 0.37, 95% CI, 0.17–0.81, *p* = 0.01) but not premenopausal ER+ breast cancer (HR 0.84, 95% CI, 0.63–1.14, *p* = 0.27) [56]. The data are influenced by the predominance of ER+/PR+ postmenopausal breast cancer, the relative frequency of ER+ vs. ER- breast cancers and the lack of BMS intervention studies that include the menopausal and ER+/ER- breast cancer status [74,83,109,110].

Furthermore, the Reach for Health study demonstrated that improved glycaemic control achieved via metformin therapy and weight loss had a positive impact on the plasma insulin, SHBG and oestrogen concentrations in postmenopausal breast cancer survivors who were either overweight or obese [111]. These improved biomarkers were associated with a reduced risk of recurrence and cancer-related mortality, supporting the preventative and prognostic effect of bariatric surgery and weight loss for female-specific and oestrogen-sensitive cancers such as breast, endometrial and ovarian cancer [107,108,109,110].

#### 5.2.2. HCC

Previous epidemiological studies have shown increasing BMI has a linear relationship with the development of NASH/NAFLD, with a five to nine-fold increase at a BMI of 30–32.5 kg/m^2^ and a ten to fourteen-fold increase at a BMI of 37.5–40 kg/m^2^ compared to patients with a normal BMI (20–22.5 kg/m^2^) [112]. NASH/NAFLD is a risk factor for the development of cirrhosis (HR 4.73, 95% CI 2.43–9.19) and HCC (HR 3.51, 95% CI 1.72–7.16) compared to matched controls. Diabetes is an independent risk factor for the progression of NASH/NAFLD (and also matched controls) to cirrhosis or HCC [113]. There was a significant reduction in the incidence of HCC after bariatric surgery in our subgroup meta-analysis (RR 0.35, 95% CI 0.22–0.55, *p* < 0.00001). This correlates with a recent histology-based study of NASH regression in obese patients after BMS, which demonstrated 29% of patients had normal liver histology at the follow-up biopsy (>12 months postoperatively), 74% had NASH resolution without fibrosis progression and 70% had ≥1 stage fibrosis regression. This was associated with successful weight loss and improvements in the risk factors of diabetes and hypertension in patients following bariatric surgery. Older age, lower weight loss and failure of resolution of the metabolic syndrome were significant risk factors for persistent advanced hepatic fibrosis [114]. Furthermore, a 2021 systematic review and meta-analysis of nine studies involving patients with NAFLD/NASH reported a reduction in HCC incidence following bariatric surgery in an adjusted model derived from matched cohorts (OR 0.63; 95% CI: 0.53–0.75) [115]. 

#### 5.2.3. Colorectal Cancer

Our meta-analysis demonstrated a decrease in the overall colorectal cancer incidence (RR 0.69, 95% CI 0.53–0.88, *p* = 0.003). After the sensitivity analysis and removal of one study that was a significant outlier, the risk reduction was similar between males (RR 0.59, 95% CI 0.40–0.87, *p* = 0.008) and females (RR 0.61, 95% CI 0.44–0.84, *p* = 0.002), with a lower overall CRC risk (RR 0.63, CI 0.50–0.81, *p* = 0.0002). Previous studies have demonstrated considerable variability in the future risk of CRC following bariatric surgery, with some reporting no difference [59], a protective effect [55,57] or an elevated risk of CRC, particularly in male patients or after RYGB surgery [33,45,58,63]. This disparity may be related to differences in the study design, including the specific type of bariatric surgery and younger age of bariatric surgery patients compared to some population-based control cohorts, using a population-based standardised incidence ratio (SIR) rather than matched cohort control patients for cancer incidence or short follow-up time. This is due to confounding or selection bias when cohorts are not properly matched for age, BMI, alcohol intake, smoking status or diabetes [59]. There may be sexual dimorphism in the intestinal estrolobiome, dietary phytonutrient and carcinogen intake and resolution of the metabolic syndrome after bariatric surgery. Changes in the bile acid pool, increased GLP-1 release, colorectal mucosal COX-1 expression and rectal mucosal proliferation after RYGB have also been proposed, but further data are required [33,38,45,58,59,60,61,62,63].

#### 5.2.4. Pancreatic Cancer

A significantly decreased risk of pancreatic adenocarcinoma incidence (RR 0.52, 95% CI 0.29–0.93, *p* = 0.03) was found after pooling seven studies of pancreatic cancer incidence after bariatric surgery. Recognised risk factors from previous epidemiological studies in pancreatic cancer include cigarette smoking, central obesity, T2DM, chronic pancreatitis, a high-fat diet, red meat consumption and excessive alcohol intake, with a slightly higher incidence in males [116]. In particular, visceral obesity, a high-fat diet, T2DM, IGF release and hyperinsulinemia are associated with pancreatic duct cell proliferation, pancreatic stellate cell activation, TGF-β1 release, pancreatic fibrosis and KRAS activation. A diet rich in fruits, vegetables and dietary fibre is thought to be protective against the development of pancreatic cancer. Improvements in chronic peripancreatic adipose tissue inflammation, leptin, hyperinsulinemia, T2DM, KRAS activation, IGF and intestinal microbiome after dietary intervention and bariatric surgery may explain the decreased development of pancreatic ductal adenocarcinoma after bariatric surgery [116].

#### 5.2.5. Gallbladder Cancer

Gallbladder cancer, but not extrahepatic bile duct cancer, was identified by the IARC in 2016 as an OAC [8]. The pathogenesis of gallbladder cancer is linked to chronic cholelithiasis under the influence of oestrogen and altered biliary excretion of cholesterol, particularly with ageing and females with overweight or obesity [117]. A previous meta-analysis of prospective observational studies found each 5 kg/m^2^ increase in BMI in women was associated with a 59% increased risk of gallbladder cancer [117]. Demonstrating a benefit of bariatric surgery has proven elusive due to the comparatively low incidence of gall bladder cancer and the small number of relevant studies. In our present meta-analysis, bariatric surgery was found to be protective against future gallbladder cancer risk (RR 0.41, 95% CI 0.18–0.96, *p* = 0.04).

#### 5.2.6. Gastric and Oesophageal Cancer

There was no significant decrease in gastric or oesophageal cancer after bariatric surgery in the present meta-analysis. Previous epidemiological studies have shown that oesophageal adenocarcinoma is associated with male sex, cigarette smoking, central obesity, metabolic syndrome, gastro-oesophageal reflux, Barrett’s oesophagus and dietary risk factors [2,8,10]. In 2016, the IARC reported an increased relative risk of 4.8 (95% CI, 3.0–7.7) of oesophageal adenocarcinoma and 1.8 (95% CI,1.8–2.5) in cardia gastric cancer in patients with BMI > 40 kg/m^2^ [8]. Gastric cardia and oesophageal adenocarcinoma share many of the same risk factors, and as such, there has been controversy over whether reflux-inducing bariatric operations such as sleeve gastrectomy or duodenal switch may contribute to this risk, as opposed to Roux-en-Y gastric bypass surgery. An increased risk of oesophageal adenocarcinoma after BMS has not been shown in recent large population-based studies [39,43]; however, it was suggested that a properly powered study with an adequate 10-year follow-up period would require over 34,000 LSG and RYGB procedures in each arm [64]. Non-cardia gastric cancer is not recognised as an OAC, and its most important risk factor is *Helicobacter pylori* chronic gastritis [2,3,8]. This is the reason why routine endoscopy and *H. pylori* eradication prior to bariatric surgery has been recommended to prevent gastric cancer development in the gastric remnant. Limitations in analysing the existing gastric cancer studies include the lack of separation of cardia and non-cardia gastric cancer data (with the exception of Aminian et al. 2022 and Lazzati et al. 2022), as well as the female preponderance in bariatric surgery data and relatively low frequency of events [38,39]. 

#### 5.2.7. Cancer-Associated Mortality

There was a significant reduction in the overall cancer-associated mortality (RR 0.51, 95% CI 0.42–0.62, *p* < 0.00001) after bariatric surgery in this meta-analysis. This correlates with previous research studies that reported increased cancer progression and recurrence, poorer cancer prognosis, the development of second primary cancers and impaired quality of life in patients with obesity [118,119,120,121]. Both Aminian et al. (2022) and Schauer et al. (2017) observed a significant ‘dose-dependent’ relationship with weight loss after bariatric surgery on cancer risk and survivorship at ten years [38,101]. This indicates the importance of designing, implementing and continuing studies of obesity interventions with longer-term follow-up, as well as the close relationship between the genesis and potentiation of cancer and the metabolic syndrome [109]. Much of the data on cancer survivorship are based on breast or colorectal cancer studies, indicating the need to investigate outcomes for a wider range of OACs [118].

### 5.3. Limitations

There are some key limitations to the conclusion from this SRMA that future cancer risk is reduced after bariatric surgery compared to conventional management. Firstly, all included studies were retrospective cohort studies apart from the three studies analysing the results from the SOS trial, which was a prospective controlled intervention study [48,53,59,122,123]. This is due to the cost, feasibility and ethical considerations of conducting prospective, randomised controlled trials in the study population. The use of observational studies introduces many potential confounders, which is important when evaluating association and causation according to the Bradford Hill epidemiological criteria [124]. The confounding factors were evaluated using the ROBINS-I for risk of bias (ROB). The key confounders included patient age, sex, country of trial, duration of follow up, comorbidities, smoking history and alcohol consumption. Missing information on these variables and other key aspects, such as the BMI, resulted in an elevated risk of overall bias, as well as potential residual bias, across the studies. The electronic health records and databases used can be prone to misclassification and coding errors, which can compromise the accuracy of the data [38]. Many of the included studies may include a ‘healthy user bias’, since patients that receive bariatric surgery are more likely to adopt positive lifestyle changes such as smoking cessation and dietary and exercise modifications during follow-up [51]. It has been proposed that surveillance bias occurs in the bariatric surgery group, since patients are more likely to have had cancer screening tests performed than their nonsurgical counterparts in both the postoperative and nonoperative periods [38]. Selection bias may have been a factor, with Chao (2021) noting that the eligibility criteria for bariatric surgery such as smoking cessation may result in surgical patients having a lower cancer risk than the nonsurgical control group [124]. Other potential sources of confounding or selection bias include clinically eligible patients with morbid obesity who were unable to access bariatric surgery due to a low socioeconomic status or geographic remoteness [68,125].

A further potential bias was the lack of long-term follow-up across some of the studies [33,106]. Aminian et al. (2022) reported a separation in the Kaplan–Meier curves for obesity-associated cancer incidence between BMS and no surgery after six years of follow-up [38]. However, some of the included studies in our meta-analysis have shorter follow-up periods (<5 years), which may have resulted in lower incidence rates for certain cancers, limiting the statistical power and increasing the likelihood of underestimation. Differences in age is another factor limiting the statistical power of the meta-analysis, since the mean age of patients who received bariatric surgery was approximately 44 years and 46 years in the control group of all included studies. This is because the inclusion of younger-aged patients in conjunction with short-term follow-up may lead to a lower incidence of age-related cancers such as colorectal, prostate, gall bladder, oesophageal adenocarcinoma or postmenopausal breast cancer [38,61].

The types of procedures performed across the studies may also affect the SRMA results. RYGB and LSG have been performed more frequently than other types of bariatric surgery (LAGB and VBG) since 2007 [77]. RYGB is usually offered to individuals with higher BMIs and is associated with greater weight loss and long-term metabolic effects than restrictive procedures such as LAGB, which may act as a confounding variable [30,101]. A breakdown of different bariatric procedures was not always provided, which means that other covariables such as the BMI and percent loss of the TBW become more important for correlation with the cancer risk.

There are also limitations in the systematic review process, in addition to those present in the included studies. For instance, it is possible that our literature search did not capture all relevant studies between 2007 and 2023. However, the reference lists of relevant studies were reviewed for studies that were not identified in the initial search, and the inclusion or exclusion of studies was discussed between two reviewers.

### 5.4. Implications for Clinical Practice and Future Directions 

This systematic review and meta-analysis have suggested a protective effect of bariatric surgery, with risk reductions for the overall cancer incidence, obesity-associated cancer incidence and cancer-related mortality. Risk reductions were particularly notable for hormone-sensitive, female-specific cancer incidences, including breast cancer, endometrial cancer and ovarian cancer. Significant risk reductions for pancreatic, gall bladder, hepatocellular carcinoma and colorectal cancer incidences were also observed. These findings confirm the effect of bariatric surgery on the future cancer risk and cancer-related mortality in obese patients. 

However, all studies included in this review were observational in nature, indicating the need for more prospective research to be conducted on the impact of bariatric surgery on cancer risk. Longer-term follow-up is required to investigate the ‘dose-dependent’ response of bariatric surgery on the cancer risk and survivorship. In addition, an extended period of follow-up would enable the exploration of weight loss trajectories following bariatric surgery and an evaluation of the incidence and effect of weight regain on cancer risk. 

Recent randomised placebo-controlled medical trials of a long-term, once-weekly s/c GLP-1 receptor agonist (STEP 1 trial, semaglutide, 2.4 mg/week) reported a mean 14.9% TBW loss at 68 weeks and once-weekly s/c GLP-1R/GIP-R co-agonists (SURMOUNT-1 tria1, tirzepatide, 15 mg/week) a 20.9% TBW loss at 72 weeks in obese patients [126,127]. A similar result was achieved with once-weekly s/c semaglutide (2.4 mg/week) in the STEP 6 RCT involving obese Japanese and South Korean patients (BMI ≥ 27 kg/m^2^) of 13.2% TBW loss at 68 weeks [128]. The observed weight loss and improvements in cardiometabolic risk factors, CRP and VAT volume are certainly promising. However, the STEP 1 extension study, which followed 228 patients who had lost 17.3% of TBW, found that one year after cessation of semaglutide and lifestyle interventions, patients regained two-thirds of the weight (11.6% TBW) they had lost [129]. The cardiometabolic improvements also reverted toward the baseline levels, including prediabetes (HbA1c), hyperlipidaemia, inflammation (CRP) and hypertension. The steepest weight regain after the cessation of semaglutide occurred in patients who had previously lost >20% of their TBW [129], which reflects the persistence of neurohormonal and behavioural pathways in patients with obesity [24]. How pharmacological treatment translates to a long-term reduction in cancer incidence in obese patients remains to be shown [126,127,128,129].

There is an opportunity for research to be conducted on the effect of bariatric surgery/weight loss on different types of OACs, especially for cancers such as CRC, where there is considerable variability in the existing data. Additional research is required on the subtypes of various cancers and specific patient populations, such as pre- and postmenopausal breast cancer and ER-negative and ER-positive breast cancer, which would enable clarity on the molecular mechanisms involved.

## 6. Conclusions

Obesity-associated carcinogenesis is closely related to metabolic syndrome; hyperinsulinemia; visceral adipose dysfunction; aromatase activity and detrimental cytokine, adipokine and exosomal miRNA release. Bariatric surgery results in long-term weight loss in patients with morbid obesity and markedly improves the features of metabolic syndrome and hormonal dysfunction. Accordingly, a substantial risk reduction after bariatric surgery has been suggested for the overall cancer-related mortality and cancer incidence in this systematic review and meta-analysis. The incidence of seven obesity-associated cancers was significantly decreased by bariatric surgery, particularly gynaecological malignancies (endometrial, ovarian and breast cancer) and hepatocellular carcinoma, gall bladder, pancreatic and colorectal cancer. This has implications for the prevention of future cancer risks associated with increasing obesity levels in the population.

## Figures and Tables

**Table 1:** Characteristics of the studies included in the meta-analysis [38,39,40,41,42,43,44,45,46,47,48,49,50,51,52,53,54,55,56,57,58,59,60,61,62,63,64,65,66,67,68,69].

Study (Author, Year)	Country of Origin, Database	Study Design	Time Period	Cancer Type	Mean Follow up Period	Sample Size	Female (%)	Mean Age Years	BMI (kg/m^2^)	Type of Bariatric Surgery
					BS NS	BS NS	BS NS	BS NS	BS NS	
Aminian 2022 [38]	USA, Cleveland Clinic Health System (CCHS)	Retrospective Institution-based cohort study	2004–2017	Overall, OAC	5.8 years (3.8–8.8) 6.1 years (3.9–8.9)	5053 25,265	3884 (76.9%) 19,514 (77.2%)	46.0 (37.0–55.0) 46.0 (34.0– 57.0)	45.5 (41.0–51.6) 45.1 (40.7–50.1)	RYGB (*n* = 3348; 66%), SG (*n* = 1705; 34%)
Lazzati 2022 [39]	France French National Programme (PMSI)	Retrospective, population-based, multicentre, cohort study	2010–2019	Overall, OAC, Non OAC	5.7 +/− 2.2 6.5 +/− 2.3 years	288,604 851,743	235,410 (81.5%) 469,746 (55.1%)	39.8 +/− 11.5 51.8 +/− 11.1	NA NA	Open or lap AGB, RYGB, SG No % provided
Khalid 2021 [40]	USA, all-Payer Claims Database, Mariner	Retrospective cohort study	2010–2018	Overall, OAC	5 years (total follow up)	19,272 9636	15,828 (82.13%) 7914 (82.13%)	NA NA	NA NA	VSG (*n* = 9636), RYGB (*n* = 9636)
Kao 2021 [41]	Taiwan, National Health Insurance Research Database (NHIRD)	Retrospective Population based cohort study	2000–2015	Overall	6.32 years 8.23 years	9250 93,880	5621 (60.77%) 52,639 (56.07%)	33.89 ± 8.82 38.56 ± 9.80	NA NA	NA
Tsui 2020 [42]	USA, New York Statewide Planning and Research Cooperative System database	Retrospective State-based cohort study	2006–2012	Overall Cancer Incidence, OAC, Non-OAC	NA NA	71,000 323,197	55,781 (78.6%) 247,102 (76.5%)	44 +/−18 45 +/−19	NA NA	RYGB SG LAGB % not provided
Tao 2020 [43]	Nordic countries-Nordic Obesity Surgery Cohort (NordOSCo) (Denmark, Finland, Iceland, Norway and Sweden), national patient registries	Retrospective population-based cohort study	1980–2012	Overall, OAC, non-OAC	NA NA	49,096 433,476	36,533 (74.4%) 318,596 (73.5%)	NA NA	42.4 40.1	RYGB (*n* = 35,541 (72.4%)), AGB VBG
Schauer 2019 [44]	USA, Kaiser Permanente	Retrospective multi-centre matched cohort study	2005–2012	Overall, breast cancer, CRC, endometrial cancer, PDAC	3.5 years 3.5 years	22,198 66,427	17,980 (81%) 53,892 (81%)	45.0 (11) 45.0 (11)	44.84 (6.71) 44.37 (6.24)	RYGB (*n* = 13,545 (61.0%)) SG (*n* = 6047 (27.2%)) LAGB (*n* = 1236 (5.6%))
Mackenzie 2018 [45]	UK, Hospital Episode Statistics database in England	National population-based cohort study	1997–2012	Overall, breast, endometrial, prostate, CRC, oesophageal cancers	55 mths median 55 mths median	8794 8794	7069 (80.4%) 7069 (80.4%)	42 (median) 42 (median)	NA NA	RYGB (*n* = 4978 (56⋅6 %)), AGB (*n* = 2957 (33⋅6%)) SG (*n* = 859 (9⋅8 %))
Pontiroli 2018 [46]	Italy, Regional Lumbardy Administrative Database	Retrospective region-based cohort study	1988–2018	Overall	19.5 ± 1.87 years 19.5 ± 1.87 years	154 360	NA NA	31.8 ± 6.43 51.8 ± 5.89	42.7 ± 4.62 39.1 ± 5.27	LAGB
Douglas 2015 [47]	UK, UK Clinical Practice Research Datalink (CPRD)	Retrospective cross-sectional population-based cohort study	CPRD records to the end of 2014	Overall	3.4 years 3.4 years	3882 3882	3126 (81%) 3166 (82%)	45 45	44.7 42.1	Gastric bypass (*n* = 1421) SG (*n* = 613) Gastric band (n = 1829)
Sjostrom 2013 [48]	Sweden, Swedish Obese Subjects (SOS) study	Prospective, matched, surgical intervention study	1987–2001	Overall		2010 2037	1420 (70.6%) 1447 (71.0%	46.1 (5.8) 47.4 (6.1)	41.8 (4.4) 40.9 (4.3)	RYGB (*n* = 265 (13%)), AGB (*n* = 376(19%)) VBG (*n* = 1369 (68%))
Adams 2009 [49]	USA, Utah Cancer Registry (UCR)	Retrospective State-based cohort study	1984–2007	Overall, Breast cancer, CRC, melanoma, NHL, PDAC	12.5 years	6596 9442	5654 (86%) 7872 (83%)	38.9 (10.3) 39.1 (10.7)	44.9 (7.6) 47.4 (6.5)	Gastric bypass
Christou 2008 [50]	Canada, McGill University Health Centre, RAMQ database	Retrospective Institution-based cohort study	1986–2002	Overall, OAC	5 years	1035 5746	679 (65.6%) 3678 (64.0%)	45.1 (11.6) 46.7 (13.1)	NA NA	RYGB (*n* = 760 (73.4%)), VBG (*n* = 194 (18.7%)) RYBG (*n* = 60 (5.8%)), LRYGB (*n* = 21 (2.1%))
Doumouras 2020 [51]	Canada, Ontario Bariatric Network (OBN)	Retrospective population-based matched cohort study	2010–2016	Overall	4.9 years	13,679 13,679	11,202 (81.9%) 11,202 (81.9%)	45.23 (10.89) 45.49 (11.63)	47.21 (8.01) 46.70 (8.44)	Gastric bypass (*n* = 11 938 (87%)) SG (*n* = 1741 (13%))
Gribsholt 2016 [52]	Denmark, Danish National Patient Registry	Retrospective Nationwide cohort study	2006–2010	Overall	4.2 years	9895 247,366	7069 (80.4%) 7069 (80.4%)	40.2 years 40.2 years	NA NA	RYGB
Anveden 2017 [53]	Sweden, Swedish Obese Subjects (SOS) study	Prospective, matched, surgical intervention study	1987–2013	Female specific	18.1 years	1420 1447		47.2 (6.0) 48.8 (6.3)	42.8 (4.3) 40.7 (4.6)	NonAd/AGB (*n* = 260 (18.3%)), VBG (*n* = 970 (68.3%)), VBG/ gastric bypass (*n* = 190 (13.4%))
Doumouras 2022 [54]	Canada, Ontario Bariatric Network (OBN)	Retrospective population-based matched cohort study	2010–2016	Breast	3 years BS	12,724 12,724	12,724 (100%) 12,724 (100%)	45.09 (10.95) 45.09 (10.08)	NA NA	RYGB LSG No % provided
Tsui 2021 [55]	USA New York Statewide System database	Retrospective State-based matched cohort	2006–2012	Female specific cancer (breast, ovarian, endometrial)	N/A N/A	55,781 247,102	55,781 (100%) 247,102 (100%)	43.00±18.0 44.00±19.0	N/A N/A	LAGB, LSG, RYGB No % provided
Feigelson 2020 [56]	USA, Kaiser Permanente	Retrospective multi-centre cohort study	2005–2012	Breast Cancer	47.5 mths (23.2) 40.8 mths (24.7)	17,998 53,889		44.6 (11.1) 44.7 (11.0)	44.6 (6.6) 44.1 (6.2)	RYGB (61.3%), SG (27.0%)
Hassinger 2019 [57]	USA Virginia University clinical data repository	Retrospective Single institution-based propensity matched cohort	1985–2015	Breast	6.5 +/− 6.1 6.3 +/− 5.0 years	2430 2430	2430 (100%) 2430 (100%)	42 48 *p* < 0.001	48.7 39.2 *p* < 0.001	RYGB, 79.4% SG, 7.5% AGB, 11.9% Other, 1.2%
Hussan 2022 [58]	USA, Market Scan database	Retrospective cohort study	2012–2020	CRC	3.1 (2.1) years 3.0 (2.1) years	88,630 327,734	68,766 (77.6%) 250,564 (76.5%)	43.4 (10.8) 43.7 (11.0)	NA NA	RYGB (*n* = 26,877 (30.3%)), VSG (*n* = 61,753 (69.7%))
Taube 2021 [59]	Sweden Swedish Obese Subjects (SOS) Study	Prospective non- randomized matched cohort	1987–2001	CRC	22.2 years	2006 2038	1419 (70.7%) 1445 (70.9%)	47.2 ± 5.9 48.7 ± 6.3 *p* < 0.001	42.4±4.5 40.1±4.7 *p* < 0.001	18.7% AGB 68% VBG 13.2% RYGB
Bailly 2020 [60]	France, French national health insurance information system database	Retrospective, population-based, multicentre, cohort study	2009–2018	CRC	5.7 (2.2) years 5.3 (2.1) years	74,131 971,217	57,800 (78.0%) 479,965 (49.4%)	57.3 (5.5) 63.4 (7.0)	NA NA	AGB, SG, RYGB
Kwak 2019 [61]	USA, University of Virginia Database	Retrospective institution-based cohort study	1985–2015	CRC	7.8 years	2231 2231	1,846 (82.7%) 1,882 (84.4%)	42.6 (10.3) 42.8 (13.4)	48.3 (8) 49.3 (11.4)	RYGB (77.1%) AGB (13.6%) SG (7.4%) Other (2%)
Aravani 2018 [62]	UK, Hospital Episode Statistics (HES) dataset	Retrospective population- based cohort study	1997–2013	CRC and OACs	3 years 2.5 years	39,747 962,860	76.60% 62.90%	44.8 53.1	NA NA	Restrictive surgery 52% Restrictive and malabsorptive surgery 48%
Derogar 2013 [63]	Sweden, Swedish Cancer Register	Retrospective register-based cohort study	1980–2009	CRC	10 years 7 years	15,095 62,016	11,608 (77%) 38,870 (63%)	39 years 49 years	NA NA	VBG (25%) AGB (24%) RYGB (51%)
Andalib 2020 [64]	Canada, 2 main provincial healthcare databases:	Retrospective population-based comparative cohort study	2006–2012	Oesophageal	7.6 years	4973 12,159	3449 (69.35% 8373 (69.80%)	RYGB 42.8 SG/DS 44.4 44.4 (10.8)	NA NA	Reflux-protective (RYGB) *n* = 852 Reflux-prone (SG & DS) *n* = 4121
Maret-Ouda 2015 [65]	Sweden, Swedish Patient Registry	Nationwide register-based cohort study	1980–2012	Oesophageal	3.7 (1.8–9.7) 3.5 (1.3–7.3) years	34,437 123,695	76% 67%	40 (33–48) 43 (32–54)	NA NA	Gastric bypass (*n* = 25,536; 74%), VBG (*n* = 4889; 14%) Gastric banding (*n* = 4012; 12%)
Rustgi 2021 [66]	USA, Truven MarketScan database	Retrospective Nationwide insurance claim-based cohort study	2007–2017	Liver and OAC	22.42 months (21.15)	33,435 64,655	24,665 (73.77%) 40,266 (62.28%)	44.01 (10.38) 45.93 (11.21)	NA NA	Lap RYGB (*n* = 12,663 (37.87%)) LSG (*n* = 11,420) (34.16%) LAGB (*n* = 4788 (14.32%) BPD-DS (*n* = 3221) (9.63%) Open RYGB (*n* = 733) (2.19%) Open VBG/SG (*n* = 272) (0.81%)
Kwak 2020 [67]	USA, University of Virginia clinical data repository (CDR)	Retrospective Institution-based cohort study	1985–2015	Liver	7.1 years	2057 2055	1709 (83%) 1783 (85%)	(median) 42 43	47 46.4	SG (*n* = 121) (5.9%), RYBG (*n* = 1617 (79%), laparoscopic gastric banding (*n* = 275 (13%) other bariatric procedures. (*n* = 44) (2%)
Yang 2016 [68]	USA, UHC Clinical Data Base (CDB)	Retrospective multicentre cohort study	2011–2015	Liver	3-year study period	178,192 15,584,065	141,274 (79.3%) 8,625,915 (55.4%)	NA NA	NA NA	NA
Botero-Fonnegra 2022 [69]	USA, National (Nationwide) Inpatient Sample (NIS)	Retrospective National database study	2010–2015	Kidney	NA NA	296,041 2,004,804	235,542 (79.6%) 1,326,439 (66.2%)	51.9 54.4	NA NA	NA

## Data Availability

Dataset is available if required.

## Appendix A

**Table A1:** Overall cancer incidence RE model sensitivity analysis.

Excluded Study	Pooled RR	LCI 95%	HCI 95%	Cochran Q	*p*	I^2^	I^2^ LCI 95%	I^2^ HCI 95%
Christou 2008 [50]	0.66	0.48	0.91	1839.72	0.00000	99.40	99.28	99.50
Lazzati 2022 [39]	0.67	0.55	0.81	354.94	0.00000	96.90	95.78	97.72
Tao 2020 [43]	0.63	0.44	0.91	1848.34	0.00000	99.40	99.29	99.50
Kao 2021 [41]	0.63	0.46	0.87	1848.72	0.00000	99.40	99.29	99.50
Pontiroli 2018 [46]	0.61	0.45	0.84	1847.67	0.00000	99.40	99.29	99.50
Sjostrom 2013 [48]	0.61	0.45	0.84	1836.02	0.00000	99.40	99.28	99.50
Schauer 2019 [44]	0.61	0.44	0.84	1771.98	0.00000	99.38	99.26	99.48
Aminian 2022 [38]	0.61	0.44	0.84	1805.85	0.00000	99.39	99.27	99.49
Adams 2009 [49]	0.61	0.44	0.83	1805.34	0.00000	99.39	99.27	99.49
Khalid 2021 [40]	0.61	0.44	0.83	1774.79	0.00000	99.38	99.26	99.48
Tsui 2020 [42]	0.60	0.44	0.81	1303.05	0.00000	99.16	98.97	99.31
Douglas 2015 [47]	0.60	0.44	0.82	1816.47	0.00000	99.39	99.28	99.49
Rustgi 2021 [66]	0.60	0.44	0.81	1502.25	0.00000	99.27	99.11	99.40

**Table A2:** Incidence of obesity-associated cancers (OAC) RE sensitivity analysis.

Excluded Study	Pooled RR	LCI 95%	HCI 95%	Cochran Q	*p*	I^2^	I^2^ LCI 95%	I^2^ HCI 95%
Lazzati 2022 [39]	0.67	0.52	0.86	144.89	0.00000	96.55	94.48	97.84
Tao 2020 [43]	0.61	0.36	1.04	847.25	0.00000	99.41	99.23	99.55
Adams 2009 [49]	0.59	0.37	0.93	844.38	0.00000	99.41	99.23	99.55
Aminian 2022 [38]	0.58	0.37	0.93	841.82	0.00000	99.41	99.23	99.54
Khalid 2021 [40]	0.57	0.35	0.91	791.08	0.00000	99.37	99.17	99.52
Rustgi 2021 [66]	0.56	0.35	0.90	802.51	0.00000	99.38	99.19	99.52
Tsui 2020 [42]	0.55	0.37	0.81	422.96	0.00000	98.82	98.35	99.15

**Table A3:** Cancer-related mortality sensitivity analysis.

Excluded Study	Pooled RR	LCI 95%	HCI 95%	Cochran Q	*p*	I^2^	I^2^ LCI 95%	I^2^ HCI 95%
Gribsholt 2016 [52]	0.53	0.43	0.65	1.13	0.77057	0.00	0.00	59.24
Doumouras 2020 [51]	0.53	0.43	0.66	1.37	0.71317	0.00	0.00	66.41
Aminian 2022 [38]	0.51	0.42	0.63	1.75	0.62541	0.00	0.00	73.78
Adams 2009 [49]	0.50	0.40	0.62	1.57	0.66730	0.00	0.00	70.65
Sjostrom 2013 [48]	0.49	0.40	0.60	0.80	0.84915	0.00	0.00	42.68

**Table A4:** Overall breast cancer random effects model sensitivity analysis.

Excluded Study	Pooled RR	LCI 95%	HCI 95%	Cochran Q	*p*	I^2^	I^2^ LCI 95%	I^2^ HCI 95%
Christou 2008 [50]	0.60	0.47	0.77	326.12	0.00000	96.63	95.37	97.54
Mackenzie 2018 [45]	0.60	0.46	0.77	311.56	0.00000	96.47	95.13	97.44
Lazzati 2022 [39]	0.58	0.46	0.73	148.83	0.00000	92.61	88.94	95.06
Tao 2020 [43]	0.57	0.43	0.74	336.49	0.00000	96.73	95.53	97.61
Doumouras 2022 [54]	0.56	0.43	0.73	340.83	0.00000	96.77	95.59	97.64
Aravani 2018 [62]	0.56	0.43	0.73	340.82	0.00000	96.77	95.59	97.64
Hassinger 2019 [57]	0.56	0.43	0.72	340.96	0.00000	96.77	95.59	97.64
Feigelson 2020 [56]	0.55	0.42	0.71	334.03	0.00000	96.71	95.49	97.60
Aminian 2022 [38]	0.54	0.42	0.70	336.75	0.00000	96.73	95.53	97.61
Anveden 2017 [53]	0.54	0.42	0.70	336.57	0.00000	96.73	95.53	97.61
Khalid 2021 [40]	0.54	0.41	0.70	322.89	0.00000	96.59	95.32	97.52
Tsui 2021 [55]	0.54	0.43	0.67	145.25	0.00000	92.43	88.64	94.95
Adams 2009 [49]	0.53	0.41	0.69	327.47	0.00000	96.64	95.39	97.55

**Table A5:** Premenopausal breast cancer random effects model sensitivity analysis.

Excluded Study	Pooled RR	LCI 95%	HCI 95%	Cochran Q	*p*	I^2^	I^2^ LCI 95%	I^2^ HCI 95%
Lazzati 2022 [39]	0.970497	0.76	1.24	2.66	0.26404	24.90618	0.00	92.19
Feigelson 2020 [56]	0.946907	0.71	1.26	5.21	0.07386	61.62083	0.00	89.05
Adams 2009 [49]	0.842874	0.71	1.00	3.32	0.19019	39.75017	0.00	81.41
Aminian 2022 [38]	0.809587	0.75	0.88	2.08	0.35300	3.96464	0.00	90.01

**Table A6:** Postmenopausal breast cancer random effects model sensitivity analysis.

Excluded Study	Pooled RR	LCI 95%	HCI 95%	Cochran Q	*p*	I^2^	I^2^ LCI 95%	I^2^ HCI 95%
Lazzati 2022 [39]	0.64	0.52	0.79	1.34	0.51073	0.00	0.00	84.52
Aminian 2022 [38]	0.43	0.14	1.31	117.21	0.00000	98.29	96.93	99.05
Feigelson 2020 [56]	0.42	0.13	1.33	65.62	0.00000	96.95	93.84	98.49
Adams 2009 [49]	0.38	0.13	1.09	107.98	0.00000	98.15	96.61	98.99

**Table A7:** Endometrial cancer random effects model sensitivity analysis.

Excluded Study	Pooled RR	LCI 95%	HCI 95%	Cochran Q	*p*	I^2^	I^2^ LCI 95%	I^2^ HCI 95%
Adams 2009 [49]	0.41	0.27	0.62	102.89	0.00000	94.17	90.34	96.48
Mackenzie 2018 [45]	0.41	0.27	0.61	103.03	0.00000	94.18	90.35	96.48
Lazzati 2022 [39]	0.42	0.31	0.56	32.61	0.00001	81.60	63.05	90.84
Aminian 2022 [38]	0.38	0.25	0.58	109.36	0.00000	94.51	90.99	96.66
Tao 2020 [43]	0.36	0.23	0.59	106.24	0.00000	94.35	90.69	96.58
Anveden 2017 [53]	0.35	0.23	0.54	106.94	0.00000	94.39	90.76	96.59
Tsui 2021 [55]	0.34	0.24	0.50	47.77	0.00000	87.44	76.43	93.31
Christou 2008 [50]	0.36	0.24	0.53	108.27	0.00000	94.46	90.89	96.63

**Table A8:** Ovarian cancer random effects model sensitivity analysis.

Excluded Study	Pooled RR	LCI 95%	HCI 95%	Cochran Q	*p*	I^2^	I^2^ LCI 95%	I^2^ HCI 95%
Aminian 2022 [38]	0.46	0.32	0.67	17.82	0.00134	77.55	45.83	90.70
Lazzati 2022 [39]	0.52	0.38	0.71	6.09	0.19250	34.32	0.00	75.25
Khalid 2021 [40]	0.46	0.30	0.72	18.13	0.00117	77.93	46.93	90.82
Adams 2009 [49]	0.44	0.29	0.65	19.02	0.00078	78.97	49.92	91.17
Anveden 2017 [53]	0.43	0.29	0.65	18.94	0.00081	78.88	49.67	91.14
Tsui 2021 [55]	0.36	0.30	0.44	2.72	0.60547	0.00	0.00	69.43

**Table A9:** Colorectal cancer random effects model sensitivity analysis.

Excluded Study	Pooled RR	LCI 95%	HCI 95%	Cochran Q	*p*	I^2^	I^2^ LCI 95%	I^2^ HCI 95%
Christou 2008 [50]	0.70	0.54	0.90	138.06	0.00000	92.03	87.97	94.72
Aravani 2018 [62]	0.74	0.57	0.95	124.28	0.00000	91.15	86.46	94.21
Tao 2020 [43]	0.72	0.55	0.95	125.48	0.00000	91.23	86.61	94.26
Bailly 2020 [60]	0.72	0.55	0.94	92.20	0.00000	88.07	81.04	92.49
Khalid 2021 [40]	0.69	0.53	0.91	138.63	0.00000	92.07	88.03	94.74
Adams 2009 [49]	0.69	0.53	0.89	138.10	0.00000	92.03	87.97	94.72
Derogar 2013 [63]	0.68	0.52	0.89	132.95	0.00000	91.73	87.45	94.55
Tsui 2020 [42]	0.67	0.52	0.88	105.14	0.00000	89.54	83.66	93.30
Kwak 2019 [61]	0.68	0.53	0.88	138.32	0.00000	92.05	88.00	94.73
Taube 2021 [59]	0.67	0.52	0.87	132.46	0.00000	91.70	87.40	94.53
Aminian 2022 [38]	0.67	0.52	0.87	135.41	0.00000	91.88	87.71	94.63
Hussan 2022 [58]	0.66	0.51	0.85	115.06	0.00000	90.44	85.24	93.81
Mackenzie 2018 [45]	0.63	0.50	0.81	118.56	0.00000	90.72	85.73	93.97

**Table A10:** CRC male random effects model sensitivity analysis.

Excluded Study	Pooled RR	LCI 95%	HCI 95%	Cochran Q	*p*	I^2^	I^2^ LCI 95%	I^2^ HCI 95%
Aravani 2018 [62]	0.79	0.50	1.24	11.51	0.00928	73.93	27.05	90.68
Bailly 2020 [60]	0.75	0.40	1.38	13.08	0.00447	77.06	37.47	91.58
Derogar 2013 [63]	0.60	0.38	0.95	10.24	0.01663	70.70	16.20	89.76
Mackenzie 2018 [45]	0.60	0.40	0.89	12.55	0.00571	76.10	34.31	91.31
Taube 2021 [59]	0.62	0.39	0.98	13.85	0.00312	78.33	41.66	91.95

**Table A11:** CRC female random effects model sensitivity analysis.

Excluded Study	Pooled RR	LCI 95%	HCI 95%	Cochran Q	*p*	I^2^	I^2^ LCI 95%	I^2^ HCI 95%
Aravani 2018 [62]	0.89	0.54	1.49	27.24	0.00001	88.99	74.48	95.25
Bailly 2020 [60]	0.86	0.47	1.59	23.22	0.00004	87.08	68.98	94.62
Derogar 2013 [63]	0.75	0.44	1.28	26.11	0.00001	88.51	73.12	95.09
Mackenzie 2018 [45]	0.60	0.44	0.82	13.74	0.00328	78.16	41.11	91.90
Taube 2021 [59]	0.72	0.44	1.16	26.74	0.00001	88.78	73.89	95.18

**Table A12:** Oesophageal random effects model sensitivity analysis.

Excluded Study	Pooled RR	LCI 95%	HCI 95%	Cochran Q	*p*	I^2^	I^2^ LCI 95%	I^2^ HCI 95%
Lazzati 2022 [39]	0.79	0.49	1.28	15.21	0.03345	53.96	0.00	79.24
Tao 2020 [43]	0.74	0.33	1.65	51.54	0.00000	86.42	75.31	92.53
Mackenzie 2018 [45]	0.69	0.33	1.44	52.07	0.00000	86.56	75.60	92.59
Maret-Ouda 2015 [65]	0.70	0.32	1.50	51.89	0.00000	86.51	75.50	92.57
Tsui 2020 [42]	0.66	0.30	1.47	41.25	0.00000	83.03	67.98	91.00
Khalid 2021 [40]	0.65	0.32	1.31	51.68	0.00000	86.46	75.39	92.55
Adams 2009 [49]	0.63	0.31	1.30	50.83	0.00000	86.23	74.91	92.44
Aminian 2022 [38]	0.60	0.30	1.21	48.95	0.00000	85.70	73.78	92.20
Andalib 2020 [64]	0.53	0.28	1.01	38.35	0.00000	81.75	65.14	90.44

**Table A13:** HCC random effects model sensitivity analysis.

Excluded Study	Pooled RR	LCI 95%	HCI 95%	Cochran Q	*p*	I^2^	I^2^ LCI 95%	I^2^ HCI 95%
Lazzati 2022 [39]	0.43	0.32	0.57	13.37	0.03752	55.12	0.00	80.76
Kwak 2020 [67]	0.36	0.23	0.58	59.01	0.00000	89.83	81.59	94.38
Tao 2020 [43]	0.37	0.22	0.63	57.42	0.00000	89.55	80.99	94.25
Yang 2016 [68]	0.35	0.17	0.69	54.05	0.00000	88.90	79.60	93.96
Khalid 2021 [40]	0.33	0.19	0.55	58.63	0.00000	89.77	81.45	94.35
Aminian 2022 [38]	0.33	0.20	0.54	59.23	0.00000	89.87	81.67	94.40
Tsui 2020 [42]	0.31	0.18	0.53	48.61	0.00000	87.66	76.90	93.40
Adams 2009 [49]	0.34	0.21	0.53	58.89	0.00000	89.81	81.55	94.37

**Table A14:** Kidney cancer random effects model sensitivity analysis.

Excluded Study	Pooled RR	LCI 95%	HCI 95%	Cochran Q	*p*	I^2^	I^2^ LCI 95%	I^2^ HCI 95%
Lazzati 2022 [39]	0.80	0.75	0.86	7.21	0.40685	2.98	0.00	68.54
Christou 2008 [50]	0.69	0.47	1.01	187.51	0.00000	96.27	94.38	97.52
Aminian 2022 [38]	0.69	0.46	1.02	187.60	0.00000	96.27	94.38	97.52
Aravani 2018 [62]	0.69	0.46	1.04	187.60	0.00000	96.27	94.38	97.52
Khalid 2021 [40]	0.68	0.45	1.02	187.44	0.00000	96.27	94.38	97.52
Tao 2020 [43]	0.67	0.44	1.03	185.65	0.00000	96.23	94.32	97.50
Botero-Fonnegra 2022 [69]	0.68	0.41	1.11	153.98	0.00000	95.45	92.98	97.06
Tsui 2020 [42]	0.65	0.42	1.01	165.17	0.00000	95.76	93.52	97.23
Adams 2009 [49]	0.65	0.44	0.96	185.47	0.00000	96.23	94.31	97.50

**Table A15:** Gallbladder cancer random effects model sensitivity analysis.

Excluded Study	Pooled RR	LCI 95%	HCI 95%	Cochran Q	*p*	I^2^	I^2^ LCI 95%	I^2^ HCI 95%
Lazzati 2022 [39]	0.64	0.33	1.23	8.47	0.07569	52.80	0.00	82.64
Adams 2009 [49]	0.42	0.17	1.03	18.23	0.00111	78.06	47.30	90.87
Khalid 2021 [40]	0.42	0.16	1.08	17.84	0.00133	77.58	45.91	90.71
Tao 2020 [43]	0.35	0.09	1.35	14.81	0.00512	72.99	32.40	89.21
Aminian 2022 [38]	0.40	0.16	1.00	18.50	0.00098	78.38	48.23	90.97
Tsui 2020 [42]	0.30	0.14	0.63	5.27	0.26093	24.06	0.00	69.10

**Table A16:** Pancreatic cancer random effects model sensitivity analysis.

Excluded Study	Pooled RR	LCI 95%	HCI 95%	Cochran Q	*p*	I^2^	I^2^ LCI 95%	I^2^ HCI 95%
Lazzati 2022 [39]	0.63	0.45	0.88	9.53	0.08957	47.56	0.00	79.22
Christou 2008 [50]	0.54	0.30	0.99	68.32	0.00000	92.68	86.80	95.94
Tao 2020 [43]	0.55	0.26	1.15	67.15	0.00000	92.55	86.53	95.88
Khalid 2021 [40]	0.52	0.28	0.98	67.71	0.00000	92.62	86.66	95.91
Aminian 2022 [38]	0.51	0.27	0.94	67.21	0.00000	92.56	86.55	95.89
Tsui 2020 [42]	0.48	0.26	0.89	34.13	0.00000	85.35	70.03	92.84
Adams 2009 [49]	0.44	0.24	0.80	59.41	0.00000	91.58	84.45	95.45

**Table A17:** Multiple myeloma random effects model sensitivity analysis.

Excluded Study	Pooled RR	LCI 95%	HCI 95%	Cochran Q	*p*	I^2^	I^2^ LCI 95%	I^2^ HCI 95%
Lazzati 2022 [39]	0.71	0.48	1.06	7.75	0.17049	35.49	0.00	74.21
Christou 2008 [50]	0.55	0.26	1.17	53.43	0.00000	90.64	82.38	95.03
Tao 2020 [43]	0.59	0.25	1.39	52.26	0.00000	90.43	81.91	94.94
Adams 2009 [49]	0.52	0.24	1.14	53.34	0.00000	90.63	82.34	95.02
Khalid 2021 [40]	0.51	0.22	1.17	52.86	0.00000	90.54	82.16	94.99
Tsui 2020 [42]	0.47	0.23	0.94	20.04	0.00123	75.04	43.51	88.97
Aminian 2022 [38]	0.47	0.21	1.05	50.77	0.00000	90.15	81.29	94.82

**Table A18:** Thyroid cancer random effects model sensitivity analysis.

Excluded Study	Pooled RR	LCI 95%	HCI 95%	Cochran Q	*p*	I^2^	I^2^ LCI 95%	I^2^ HCI 95%
Tao 2020 [43]	0.90	0.69	1.17	22.88	0.00013	82.52	59.91	92.38
Lazzati 2022 [39]	0.91	0.72	1.16	8.28	0.08172	51.71	0.00	82.26
Adams 2009 [49]	0.86	0.66	1.12	25.68	0.00004	84.43	65.13	93.04
Aminian 2022 [38]	0.83	0.62	1.09	25.83	0.00003	84.51	65.36	93.08
Khalid 2021 [40]	0.82	0.61	1.10	25.71	0.00004	84.44	65.18	93.05
Tsui 2020 [42]	0.75	0.65	0.86	4.41	0.35356	9.26	0.00	81.13

**Table A19:** Gastric cancer random effects model sensitivity analysis.

Excluded Study	Pooled RR	LCI 95%	HCI 95%	Cochran Q	*p*	I^2^	I^2^ LCI 95%	I^2^ HCI 95%
Lazzati 2022 [39]	0.92	0.46	1.86	4.91	0.17881	38.85	0.00	79.14
Aminian 2022 [38]	0.64	0.21	1.97	36.81	0.00000	91.85	82.32	96.24
Tsui 2020 [42]	0.62	0.13	3.02	27.56	0.00000	89.11	74.84	95.29
Adams 2009 [49]	0.52	0.17	1.61	34.30	0.00000	91.25	80.73	96.03
Khalid 2021 [40]	0.41	0.15	1.13	18.60	0.00033	83.87	59.30	93.61

**Table A20:** Prostate cancer random effects model sensitivity analysis.

Excluded Study	Pooled RR	LCI 95%	HCI 95%	Cochran Q	*p*	I^2^	I^2^ LCI 95%	I^2^ HCI 95%
Lazzati 2022 [39]	1.25	0.79	1.96	3.17	0.20513	36.87	0.00	79.98
Aminian 2022 [38]	0.78	0.15	3.93	69.93	0.00000	97.14	94.30	98.57
Khalid 2021 [40]	0.63	0.16	2.50	50.61	0.00000	96.05	91.55	98.15
Adams 2009 [49]	0.61	0.16	2.37	53.43	0.00000	96.26	92.09	98.23
